# *ATP7B* variant c.1934T > G p.Met645Arg causes Wilson disease by promoting exon 6 skipping

**DOI:** 10.1038/s41525-020-0123-6

**Published:** 2020-04-08

**Authors:** Daniele Merico, Carl Spickett, Matthew O’Hara, Boyko Kakaradov, Amit G. Deshwar, Phil Fradkin, Shreshth Gandhi, Jiexin Gao, Solomon Grant, Ken Kron, Frank W. Schmitges, Zvi Shalev, Mark Sun, Marta Verby, Matthew Cahill, James J. Dowling, Johan Fransson, Erno Wienholds, Brendan J. Frey

**Affiliations:** 1 Deep Genomics Inc., 661 University Avenue, MaRS Centre West Tower Suite 480, Toronto, ON M5G 1M1 Canada; 2 Present Address: WuXi AppTec, East Windsor, NJ USA; 3 Present Address: Tesseraqt Optimization Inc., 222 College Street, Toronto, ON M5J 3J1 Canada

**Keywords:** Molecular medicine, Medical genetics

## Abstract

Wilson disease is a recessive genetic disorder caused by pathogenic loss-of-function variants in the *ATP7B* gene. It is characterized by disrupted copper homeostasis resulting in liver disease and/or neurological abnormalities. The variant NM_000053.3:c.1934T > G (Met645Arg) has been reported as compound heterozygous, and is highly prevalent among Wilson disease patients of Spanish descent. Accordingly, it is classified as pathogenic by leading molecular diagnostic centers. However, functional studies suggest that the amino acid change does not alter protein function, leading one ClinVar submitter to question its pathogenicity. Here, we used a minigene system and gene-edited HepG2 cells to demonstrate that c.1934T > G causes ~70% skipping of exon 6. Exon 6 skipping results in frameshift and stop-gain, leading to loss of *ATP7B* function. The elucidation of the mechanistic effect for this variant resolves any doubt about its pathogenicity and enables the development of genetic medicines for restoring correct splicing.

## Introduction

Wilson disease (OMIM #277900) is a recessive disorder caused by homozygous or compound heterozygous loss-of-function variants in the *ATP7B* gene (ATPase copper transporting beta; NCBI Gene ID: 540), with an estimated prevalence of 3.3/100,000 subjects^1^. *ATP7B* is a transmembrane copper transporter that primarily exerts its function in liver hepatocytes, where it is required for copper loading onto ceruloplasmin, the main bloodstream copper transporter, and for excess copper excretion into the bile. Insufficient *ATP7B* function results in copper accumulation in hepatocytes, which leads to liver pathology, and also accumulation in other organs such as the brain, which leads to neurological and neuropsychiatric alterations^1^. Physiologically, copper is bound by extracellular and intracellular chaperones like ceruloplasmin, whereas excess copper often exists in the chaperone-free form. Free copper causes oxidative damage to tissues and leads to Wilson disease, which is fatal if untreated^1^. While the standard of care overall improves patient life expectancy and quality of life, 45% of patients exhibit poor long-term adherence because of adverse effects or cumbersome dosing requirements, and 10% of patients with neurological symptoms deteriorate after treatment. Consequently, there is a recognized need for novel and improved therapeutics^1^.

The variant NM_000053.3:c.1934T > G (p.Met645Arg, hg19/b37 genomic coordinates chr13:52535985:A > C, dbSNP rs121907998) has been reported in several Wilson disease patients, typically in compound heterozygosity with truncating (i.e., stop-gain, frameshift, large deletions) or missense variants and only once in homozygosity. It was first identified in the Jewish ethnic group of a study comprising 183 unrelated families of diverse ethnic origin (*N* = 13 Jewish-American, *N* = 7 from Puerto Rico, *N* = 15 from Costa Rica, *N* = 9 from Sicily, *N* = 99 other from US, *N* = 22 from Sweden, *N* = 18 from Russia), with an overall frequency < 1%^2^. It was then identified in 1/3 unrelated Ashkenazi Jewish families from Israel^3^, in 2/118 alleles of patients predominantly of Italian origin^4^, in 2/17 unrelated patients from the Gran Canaria island^5^, in 1/46 unrelated Brazilian patients^6^, in 22/40 (55%) unrelated patients from Spain^7^, in 1/12 families from Galicia^8^, in 3/268 alleles of predominantly Italian descent (and specifically in Colombian or Spanish individuals)^9^, in 1/47 unrelated Italian pediatric patients^10^, in two unrelated patients of Ecuadorian and Moroccan ethnicity^11^, in 1/35 patients from Southern Brazil with predominant non-Spanish European descent^12^, in 1/26 unrelated families from Venezuela^13^, in 16/27 (59%) Spanish but not in other patients from a predominantly Central and Eastern European study (*N* = 1357 total patients)^14^ and in 6/25 (24%) unrelated families from south-eastern Spain^15^. Finally, it was reported as a pathogenic variant commonly found in Ashkenazi Jews from Israel, without detailed statistics^16^. It is noteworthy that all of the Spanish patients from the Margarit et al.^7^ study carried c.1934T > G in compound heterozygosity with another pathogenic variant and had hepatic disease; in addition, subjects with a truncating variant in trans had an earlier disease onset (5–14 years old), whereas subjects with a missense variant in trans had more variable onset (7–50 years old)^7^. The patients from Gran Canaria^5^, south-eastern Spain^15^, and Venezuela^13^ were also compound heterozygous and had liver disease. In contrast, the Brazilian subjects were reported to have both liver and neurological disease (with the latter deemed of greater severity)^6^ or only neurological disease^12^. These details were not available for other reported cases. The Italian pediatric patient was the only homozygous individual; since only selected exons were sequenced in this study, an additional pathogenic variant may have been missed^10^.

Several groups have examined the Met645Arg amino acid substitution and failed to demonstrate a significant effect on protein function. First, copper transport was studied by two different groups using complementary approaches. In Sf9 insect cells infected with a viral vector carrying the ATP7B mutant protein complementary DNA (cDNA) but lacking endogenous copper transporter activity, copper transport was quantified in microsomal vesicles. Copper uptake was normal for the Met645Arg variant, whereas most of the other pathogenic missense variants that were tested resulted in reduced uptake: 14/25 displayed no or low uptake and 10/25 displayed partially reduced uptake^17^. Second, human SV40-transformed ATP7B-null (YST) fibroblasts were transfected with a plasmid containing the mutated ATP7B-mGFP cDNA, showing that the Met645Arg mutant protein was expressed and was able to transport copper, which is consistent with previous findings^18^. In addition, vesicular trafficking was characterized by the same group, monitoring migration to the apical membrane (anterograde) in response to elevated copper and return back to the Golgi apparatus (retrograde) in response to depleted copper. Polarized hepatic WIF-B cells were infected with a viral vector carrying the cDNA of the ATP7B mutant protein fused with GFP, showing normal anterograde and retrograde trafficking for Met645Arg but not for other pathogenic missense variants^18^. Last, another group studied the variant effect on ATP7B stability mediated by COMMD1 (copper metabolism domain containing 1), which binds ATP7B and promotes its proteolytic degradation, acting as a negative regulator but also as a quality control mechanism. In HEK293T cells transfected with a plasmid containing the mutated ATP7B-flag cDNA, Met645Arg did not increase COMMD1 binding. In contrast, increased COMMD1 binding and reduced ATP7B protein expression was observed for other pathogenic missense variants^19^. Based on the studies described above, it is unlikely that the amino acid change is the cause of the observed pathogenicity. Since these studies used cDNA constructs lacking introns they could not investigate effects on splicing.

As of 17 September 2019, 6/8 ClinVar submitters have reported the variant as “pathogenic” (Integrated Genetics/Laboratory Corporation of America; Genetic Services Laboratory, University of Chicago; Illumina Clinical Services Laboratory; Fulgent Genetics; Invitae; OMIM) and 1/8 as “likely pathogenic” (Counsyl). Only 1/8 submitters has classified it as a “variant of unknown clinical significance” (VUS) (SIB, Swiss Institute of Bioinformatics), which was motivated by the negative evidence on the effect of Met645Arg on protein activity (https://www.ncbi.nlm.nih.gov/clinvar/variation/3862/).

We used a minigene system and gene-edited HepG2 cells to demonstrate that c.1934T > G causes ~70% skipping of exon 6, as suggested by in-silico analysis. Exon 6 skipping results in frameshift and stop-gain, which leads to loss of *ATP7B* function. We therefore propose that c.1934T > G should be classified as a pathogenic or likely pathogenic variant.

## Results

### Overview

We carried out a thorough in-silico analysis of the c.1934T > G variant effect, which suggested that the amino acid substitution Met645Arg can be tolerated but the variant causes >50% exon 6 skipping, resulting in frameshift and stop-gain. The in-silico splicing prediction was backed by experimental high-throughput data and was further validated in a minigene system. In addition, we used CRISPR/Cas9 to obtain one clone of HepG2 cells compound heterozygous for c.1934T > G and a large insertion, and two clones of HepG2 cells homozygous for c.1934T > G; quantitative PCR (qPCR) transcript quantification showed 15% of correctly spliced wild-type transcript level in the compound het and 31–33% in the homozygous clones. Evaluation of c.1934T > G frequency suggests that it is consistent with pathogenicity. We therefore propose that c.1934T > G should be classified as a pathogenic or likely pathogenic variant.

### In-silico analysis of c.1934T > G effect

First, we validated that NM_000053 is the most appropriate principal transcript for *ATP7B* in hepatocytes. In the ClinVar data downloaded in January 2019, NM_000053 is used for 209/210 submissions of pathogenic or likely pathogenic variants. Accordingly, based on annotation, protein domain composition and conservation evidence, NM_000053 is categorized as principal by APPRIS 2019_02.v29^20^, whereas other transcripts are categorized as alternative or minor. Manual review of junctional counts from GTEx v8^21^ revealed that ENST00000242839 comprises the best supported junctions (see Supplementary Fig. 1 and Supplementary Note 1). ENST00000242839 is almost identical to NM_000053, since they differ by only a few nucleotides at the 3ʹUTR exon 21 end. In conclusion, multiple lines of evidence support using NM_000053 as the principal transcript for *ATP7B* in hepatocytes. Therefore, we used NM_000053.3 as reference transcript throughout the paper.

The Met645 amino acid position is located immediately before a predicted transmembrane element according to UNIPROT, which is consistent with the overall protein domain diagram of ATP7B showing exon 6 encoding a flexible linker between the copper binding domains and the first transmembrane element^1^. According to the multiple genome alignments provided by the UCSC hg19 genome browser, Met645 is frequently substituted by valine, leucine, alanine and threonine in mammalians and by lysine, glutamine or aspartic acid in other vertebrates; armadillo is the only mammalian showing substitution by arginine (see Supplementary Fig. 2). Consistent with this, in-silico amino acid effect predictors return impact scores for Met645Arg that correspond to lack of deleteriousness: 0.239 for SIFT, 0.146 for PolyPhen2-HDIV, 0.119 for PolyPhen2-HVAR, −1.22 for Mutation Assessor^22–24^. In conclusion, in-silico evidence suggests that this amino acid substitution can be tolerated, or perhaps produce a modest loss of function, in line with results from functional experiments^17–19^.

Next, we assessed if c.1934T > G could alter splicing. c.1934T > G is outside of the splice site consensus region (13 bp from the donor site), but it could still impact exon recognition by altering a splicing silencer or enhancer^25^. We used a deep-neural network predictor trained to recognize exons from genomic data, and applied it to score the reference and mutant sequence to obtain a variant score. The predictor is based on a deep-learning convolutional neural network that automatically learns to identify sequence motifs important for splicing^26^. This resulted in a predicted change of exon strength corresponding to >50% exon 6 skipping. To better characterize the mechanism of the splicing alteration, we additionally analyzed putative exonic splicing enhancer (ESE) sequences from three publications (Stadler et al. 6mers^27^, Ke et al. 6mers^28^, Zhang et al. 8mers^29^). Considering a +/− 5 bp window around the variant, we found that c.1934T > G reduced the number of (overlapping) Stadler et al. ESE 6mers from 5 to 2, and the number of (overlapping) Ke et al. ESE 6mers from 5 to 1. However, we did not detect any Zhang et al. ESE 8mer in a +/− 7 bp window around the variant.

Exon skipping quantification based on the high-throughput MaPSy minigene assay^30^ was available for this variant: exon 6 inclusion was decreased to 35.5% wild-type in the in-vivo dataset and 53.8% wild-type in the in-vitro dataset, in line with in-silico predictions. Since exon 6 is out-of-frame, its skipping leads to a shifted reading frame and the introduction of a stop codon in exon 8 (TGA at c.2259, genomic coordinate chr13:52532543-52532541), which is expected to cause reduced expression via nonsense-mediated decay (NMD). The corresponding HGVS protein effect is p.Glu624ValfsTer105.

It is particularly compelling that both in-silico and experimental high-throughput data suggest a splicing alteration, leading to loss of function. This prompted us to further validate this finding experimentally and to accurately quantify the amount of residual correctly spliced transcript.

### c.1934T > G causes exon skipping in a minigene system

**Fig. 1:**
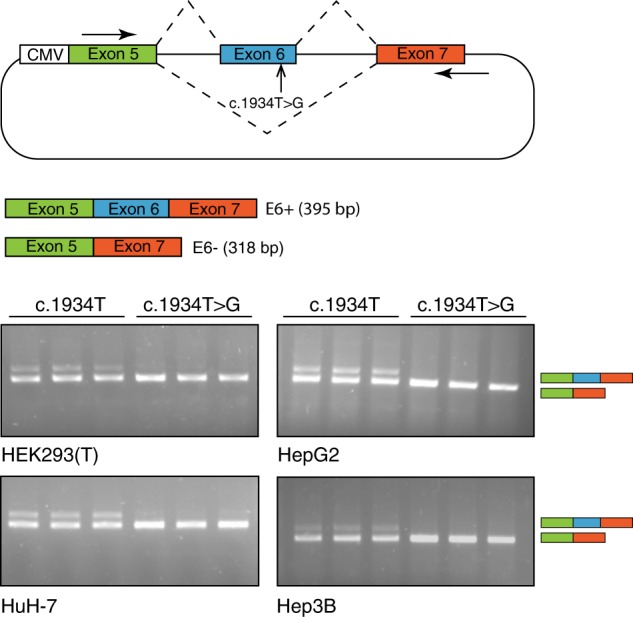
Minigene analysis of c.1934T > G variant in four different cell lines (HEK293T, HepG2, HuH-7, and Hep3B). The wild-type minigene (c.1934T) showed partial exon 6 inclusion, whereas the mutant minigene (c.1934T > G) showed almost complete skipping in all four cell lines. Experiments were performed in three separate batches of each cell type (HEK293T, HepG2, HuH-7, and Hep3B).

We constructed both a wild-type and variant-containing minigene with exon 6 flanked by full introns and partial exons 5 and 7. Splicing assays were performed in triplicate in four different cell lines by transient minigene transfection. Reverse transcription PCR (RT-PCR) showed partial exon skipping in the wild-type (c.1934T) minigene in all four cell lines (see Fig. 1), which could be caused by less-efficient splicing of minigene RNA. Importantly, however, c.1934T > G minigene had almost complete (100%) exon skipping in all cell lines and always exceeded the wild-type by a good margin, thus confirming the c.1934T > G effect on exon skipping.

### Generation of c.1934T > G edited HepG2 cells

We used CRISPR/Cas9 to obtain HepG2 cell lines with a c.1934T > G allele to study the variant effect in the endogenous gene (see Table 1). Digital droplet PCR followed by Sanger sequencing were used to screen for clones positive for the c.1934T > G variant.

**Table 1:** Edited HepG2 cells used in this study.

Clone name	Genotype
2F3	Compound het c.1934T > G, large plasmid insertion
1F6	Homozygous c.1934T > G
1E8	Homozygous c.1934T > G
2A1	Homozygous c.1931dupA (chr13:52535987:C > CT)

We first generated one clone compound heterozygous for c.1934T > G (2F3) and a complex re-arrangement. We used whole-genome sequencing (WGS) and targeted genomic PCR to carefully reconstruct its genotype: we identified a clean c.1934T > G edit on one allele, whereas the other allele presented a partial duplication of exon 6 and a ~5 kb plasmid insertion (see Supplementary Fig. 3 and Supplementary Note 1).

We additionally generated two clones homozygous for the c.1934T > G variant (1E8 and 1F6, see Supplementary Fig. 4) and one clone with a homozygous frameshift insertion (2A1, see Supplementary Fig. 5). Note that clone 2F3 was obtained using a different guide and different ssDNA repair template (ssODN) than 1E8 and 1F6 and they did not share any putative off-target site (see Supplementary Dataset 2).

### c.1934T > G causes exon skipping and reduced protein expression in edited HepG2 cells

**Fig. 2:**
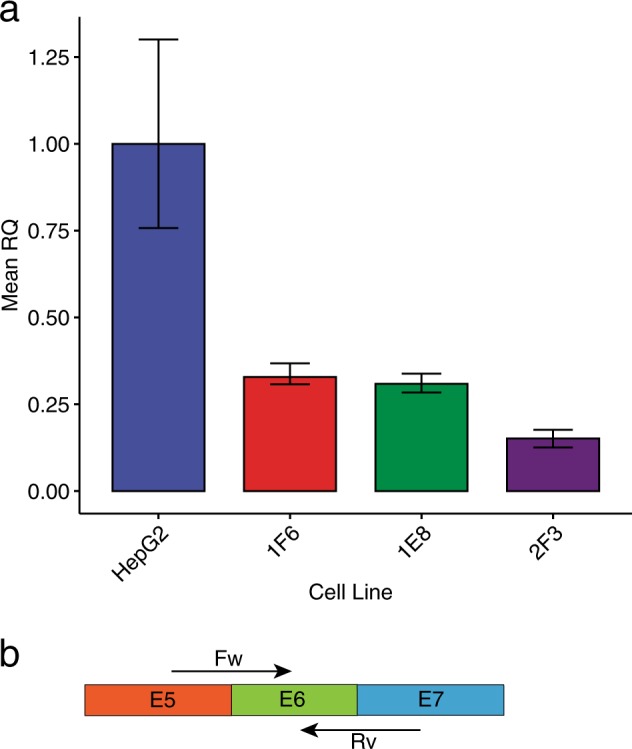
qPCR for relative quantity (RQ) of transcripts containing exons 5, 6, and 7 between wild-type HepG2, 2F3, 1F6, and 1E8 cell lines. **a** Compared to wild-type cells, 2F3 cells have only 15% of exon 5, 6, 7 spanning transcript. 1E8 and 1F6 cells have 31% and 33%, respectively, compared to wild-type cells. The barplot displays the mean RQ of 12 independent RNA extractions for each cell line, with error bars corresponding to minimum or maximum RQ. **b** PCR strategy with forward (FW) and reverse (RV) primers spanning the boundaries between exons 5 and 6, and 6 and 7, respectively.

Since exon 6 is out-of-frame, transcripts lacking exon 6 may be degraded by NMD. We thus reasoned that qPCR, with primers overlapping the exon 5–6 and exon 6–7 junctions, would be the most suitable approach to quantify the c.1934T > G splicing effect. In addition, we monitored the qPCR product length to exclude the presence of aberrant transcripts produced from the plasmid insertion allele in 2F3 cells and not degraded by NMD, which are expected to have a different length. RNA was isolated from 12 individual preparations of HepG2 wild-type, 2F3, 1F6, and 1E8 cells. First-strand synthesis and qPCR were used to calculate the relative quantity of transcripts containing exons 5, 6, and 7 between wild-type and edited cells. qPCR results showed that, compared to wild-type cells, compound heterozygous HepG2 cells (2F3 clone) express only 15% of transcript containing exons 5, 6 and 7 (see Fig. 2) and homozygous cells (clones 1E8 and 1F6) express only 31–33%. Melt curves for 2F3 cells were indicative of a single PCR product without detection of aberrant transcripts derived from the plasmid insertion (See Supplementary Fig. 6). The reduced amount of *ATP7B* transcript detected in the 2F3 clone, about a half of the homozygous clones 1E8 and 1F6, is consistent with the plasmid insertion allele not producing any stable transcript. We further confirmed by PCR that c.1934T > G causes partial skipping of exon 6 in 2F3, 1F6 and 1E8 cells (see Supplementary Fig. 7).

**Fig. 3:**
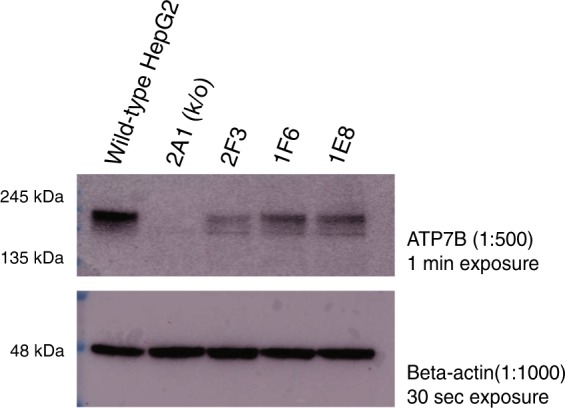
Western blot performed on total lysate(s) from wild-type and edited HepG2 cells. Immunoblot for ATP7B shows decreased protein expression in 2F3, 1F6 and 1E8 cell lines compared to HepG2 wild-type cells; protein expression in 1F6 and 1E8 homozygous lines is higher than in 2F3 compound heterozygous, as expected. No observed expression in the 2A1 knock-out (k/o) cell line. Immunoblot for beta-actin was used as a protein loading control.

In addition, protein lysates were extracted from wild-type and edited HepG2 cells to determine ATP7B protein expression by western blot. Compared to wild-type cells, compound heterozygous (2F3) and homozygous (1E8, 1F6) cells express reduced levels of ATP7B, with increased ATP7B expression in 1E8 and 1F6 homozygous cell lines compared to 2F3 cells, as expected. A control 2A1 *ATP7B* knock-out cell line has no detectable levels of ATP7B (see Fig. 3 and Supplementary Fig. 8).

To confirm that compound heterozygous 2F3 cells have impaired copper homeostasis, we treated WT and 2F3 HepG2 cells with escalating doses of copper (CuCl 100–700 µM) and detected oxidative stress by measuring fluorescence induced by CellROX Green oxidation. At higher copper doses, we observed ~2x production of reactive oxygen species (see Supplementary Fig. 9).

### c.1934T > G frequency and proposed ACMG classification

A large survey of Wilson disease in Spain reported c.1934T > G in 55% of the patients^7^, but did not report the variant frequency in the general Spanish population. The best matched gnomAD v2.1.1^31^ populations appears to be Latino, with frequency = 0.002233, followed by Southern European, with frequency = 0.0004342. A direct comparison of the allele frequency in this disease cohort (allele count = 22/80) to the gnomAD v2.1.1 allele frequency in the Latino population (allele count = 79/35376) results in a Fisher’s Exact Test two-sided *p*-value = 2.05 × 10^−38^ and OR = 169 (95% confidence interval = 94–296). However, it may be over-optimistic to use these allele frequencies for a direct case-control comparison because the population ethnicity is only partially matched. Assuming that c.1934T > G is typically pathogenic only in compound heterozygosity with other pathogenic variants, and that Wilson disease prevalence in Spain is the same as the consensus prevalence of 3.3/100,000, it is possible to estimate the c.1934T > G allele frequency in the general population of Spain as 0.002355 (see Supplementary Note 1 for detailed calculations), which is strikingly similar to the c.1934T > G Latino frequency in gnomAD v2.1.1 (0.002233).

The experimental evidence that c.1934T > G reduces the intact transcript to about 30% normal levels supports the ACMG^32^ pathogenicity evidence *PS3*, i.e., functional studies supportive of damaging effect on the gene or gene product. The frequency of c.1934T > G in Wilson disease patients compared to the general population supports the ACMG pathogenicity evidence *PS4*, i.e., prevalence in affected individuals significantly increased compared to controls, which could be downgraded to *PM* to account for residual uncertainties in ethnic composition. *PS3* and *PS4* result in the classification *pathogenic* based on the rule *≥2 Strong (PS1–PS4)*, whereas if *PS4* is replaced by *PM* the resulting classification is *likely pathogenic* based on the rule *1 Strong (PS1–PS4) and 1–2 Moderate*.

## Discussion

NM_000053.3:c.1934T > G (p.Met645Arg) has high prevalence among Wilson disease patients of Spanish and Jewish descent. It has clearly higher frequency among individuals with Wilson disease compared to the general population. The mutant Met645Arg protein has been studied in several functional assays, none of which revealed significant differences compared to the wild-type protein. Accordingly, in-silico analysis suggests that this amino acid substitution could be tolerated. This has raised some concerns about the pathogenicity of this variant. In-silico analysis and previously published high-throughput data suggest that NM_000053.3:c.1934T > G causes >50% exon 6 skipping. Since exon 6 is out-of-frame, skipping is expected to result in loss of *ATP7B* function. Minigene constructs confirmed c.1934T > G causes exon 6 skipping in four different cell lines. In addition, edited HepG2 cells compound heterozygous for c.1934T > G and a large plasmid insertion present a major reduction of *ATP7B* expression: qPCR detected 15% correctly spliced transcript and Western blot showed almost complete loss of protein expression. Accordingly, qPCR detected 31–33% correctly spliced transcript in edited HepG2 cells homozygous for c.1934T > G and western blot revealed less pronounced loss of protein expression. These results suggest that c.1934T > G causes Wilson disease chiefly by altering splicing and reducing *ATP7B* expression.

Compound heterozygous individuals typically develop hepatic disease, with early onset when a truncating variant is in trans (5–14 years old) and more variable onset with a missense variant in trans (7–50 years old)^7^. This, together with the amount of residual functional protein, suggests the variant has moderate severity. It is not clear what determines the propensity of this variant towards causing hepatic disease.

The paucity of c.1934T > G homozygotes among reported Wilson disease cases may be explained by the relatively high correctly spliced transcript in homozygotes (about 30%). It is thus possible that c.1934T > G homozygous patients have later-onset liver disease (e.g., 40–50 years old) and are less likely to be diagnosed for Wilson disease. It is also possible that homozygotes require additional risk factors (genetic or not) to develop Wilson disease, such as common hypofunctional variants impacting *ATP7B*. The only reported homozygous case belonged to a pediatric group with median age at diagnosis of 7.4 years and maximum age at diagnosis of 21 years, but an additional pathogenic variant may have been missed because not all exons were sequenced^10^. Anyway, these results suggest that c.1934T > G homozygotes are very unlikely to present a more severe or earlier onset disease than compound heterozygotes with a truncating variant.

In addition to dispelling doubts on c.1934T > G pathogenicity, the elucidation of the splicing effect enables the development of genetic medicines for splicing modulation. Steric blocking antisense oligonucleotides (SBOs) have been successfully used for splicing restoration in-vitro as well as in-vivo^33^. The chemical formulation with a phosphorothioate backbone and 2’OMe or 2’MOE modifications has proven particularly safe and effective, with, as of 2019, one FDA-approved drug (nusinersen, for spinal muscular atrophy)^34^, one approved investigational new drug (QR-110 for Leber congenital amaurosis)^35^ and one successful tailor-made clinical application^36^. Highly effective hepatocyte uptake can be mediated by GalNAc cluster conjugation^37^. The standard of care for Wilson disease presents serious adverse effects and adherence issues^1^, thus c.1934T > G homozygous and compound heterozygous patients could benefit from a novel SBO therapeutic restoring *ATP7B* exon 6 inclusion.

## Methods

### In-silico variant effect analysis

Amino acid impact predictions based on SIFT^22^, PolyPhen2-HDIV, and -HVAR^23^ and Mutation Assessor^24^ were obtained from dbNSFP version v3.5a^38^.

For splicing, we trained a deep-neural network that takes as input a genomic sequence, and predicts the presence of an exon or different types of negatives (e.g., an exon with an incorrect splice site). To construct the training dataset, we used the human reference sequence build 37 and well-supported (TSL = 1) annotated protein-coding exons in Gencode v25^39^ as positive examples. For each positive example, we constructed multiple negative examples that correspond to different missplicing outcomes. The network architecture includes convolutional and locally connected layers, and a recurrent network with long short-term memory along the length of the sequence as the final aggregation layer^26^. To counteract class imbalance during training, we created mini-batches balanced by the different categories of negatives and trained the classifier using the Adam optimizer^40^. We identified a set of optimal models by running random hyperparameter search and then evaluating performance on a held-out portion of the dataset, as well as additional, independent datasets capturing the splicing effect or splicing-mediated pathogenicity of genetic variants^30,41,42^. Finally, we used an ensemble of the top performing models for variant effect prediction.

### Minigene system

Minigene plasmids for *ATP7B* exon 6 were designed to contain part of the *ATP7B* genomic locus, comprising exons 5 to 7 and including the complete sequence of introns 5 and 6. A CMV promoter was placed upstream of exon 5. To minimize aberrant splicing of the transcribed mRNA fragments, consensus splice acceptor sequences of exon 5 were removed by deleting 9 nucleotides from the 5ʹ end of exon 5; likewise, consensus splice donor sequences of exon 7 were removed by deleting 40 nucleotides from the 3ʹ end of exon 7. Minigenes were constructed by DNA assembly of PCR fragments that were amplified from HEK293T genomic DNA using KOD Hot Start DNA polymerase (Novagen). For the wild-type minigene, the full fragment was amplified with primer pairs P461 and P463. For the mutant minigene, the c.1934T > G variant was introduced by site-directed mutagenesis of two overlapping fragments, which were amplified with primer pairs P461 and P465 and primer pairs P464 and P463, respectively. PCR fragments were cloned into a CMV-containing expression vector, linearized by PCR using primers P459 and P460, using a NEBuilder HiFi DNA Assembly Kit (New England Biolabs) according to manufacturer’s instructions. Plasmid DNA was isolated using the Presto Mini Plasmid Kit (Geneaid). Minigenes were used for splicing assays in three separate batches of each cell type (HEK293T, HepG2, HuH-7 and Hep3B). Total RNA was isolated 48 h post transfection using the Qiagen RNeasy kit. For RT-PCR analysis, first-strand synthesis was performed using high-capacity cDNA kit with 500 ng of RNA and random primers. PCR was performed with primers P243 and P472 and separated on 2% agarose gels stained with 0.05% Redsafe (FroggaBio). See Supplementary Dataset 1 for primer sequences. Gel bands in the same figure frame (corresponding to different cell lines) were derived from the same experiment and processed in parallel.

### Cell culture, maintenance, and transfection

HEK293T, HepG2, and Hep3B cell lines were purchased from ATCC. Huh-7 cells were purchased from JCRB. HEK293T cells were maintained in IMDM (Gibco) supplemented with 10% COSMIC serum (ThermoFisher), l-glutamine (2 mM) (Gibco) and Penicillin/Streptomycin (100 units/mL) (Gibco). Hep3B and HepG2 cells were maintained in DMEM 4.5 g/L glucose (10% FBS (Gibco) and Penicillin/Streptomycin). Huh-7 cells were maintained in DMEM 1 g/L glucose (10% FBS and Penicillin/Streptomycin). Cells were dissociated by incubation with TrypLE (ThermoFisher) and then equal parts media was added to the suspension. The cells were pelleted by centrifugation at 200 x *g* for 5 min and the supernatant was removed. Cells were then resuspended and diluted for correct plating densities. All cells were reverse transfected with 500 ng minigene plasmid DNA with Lipofectamine 3000 and P3000 reagent (Invitrogen) in 12-well plates. 4.5 × 10^5^ HEK293T cells were seeded per well. In all, 3 × 10^5^ cells per well were seeded for HepG2, Hep3B and Huh-7. Cells were negative to the MycoAlert Mycoplasma Detection Kit.

### Gene-editing of HepG2 cells and screening

The 2F3 edited HepG2 clone, compound heterozygous for c.1934T > G and a loss-of-function re-arrangement, was created by the Center for Commercialization of Regenerative Medicine (CCRM) (Toronto, Canada) using the CRISPR/Cas9 editing system and a pEGFP-puro plasmid for co-selection of transformants (see Supplementary Note 1, and Supplementary Dataset 2 for crRNA and template sequences). Single-cell derived clones were screened for the presence of the c.1944T > G variant by digital droplet PCR followed by Sanger sequencing. A positive clone was selected and the presence of the c.1944T > G variant was re-confirmed by Sanger sequencing at The Center for Applied Genomics (TCAG) (Toronto, Canada). For this, genomic DNA was extracted using gSYNC DNA Extraction kit (Geneaid) and quantified by nanovue (GE Healthcare). A touchdown PCR was performed on 200 ng of extracted genomic DNA with primers P488 and P650 to amplify a 759 bp fragment of *ATP7B* containing exon 6 and surrounding intronic regions. PCR was checked using UCSC in-silico PCR. PCR products were purified using the PCR cleanup kit (Geneaid) following the manufacturer’s instructions. DNA was submitted for Sanger sequencing at TCAG with forward and reverse PCR primers. Finally, the clone was selected and submitted for WGS.

The 2A1 clone, homozygous for a T insertion at the cut site resulting in complete loss of function, was also generated by CCRM and genotyped following the same methodology, except for the final WGS step.

The 1F6 and 1E8 clones (homozygous for c.1934T > G) were created using the CRISPR/Cas9 editing system at Deep Genomics. One million parental HepG2 cells were nucleofected with crRNAs/tracrRNA complex, ssODN and Cas9 endonuclease (IDT) using the Lonza Nucleofector 4D platform according to the manufacturer’s recommended protocol (cell line solution SF and program EH-100). Oxford Nanopore MinION sequencing was performed to verify editing efficiency. Cells were then diluted and plated at dilution in 96-well plates to isolate individual clones. Individual clones were screened by Sanger sequencing to identify edited populations. These were further confirmed by sequencing of amplicons on the MiSeq platform: a 154 bp genomic DNA fragment surrounding the variant knock-in site was PCR-amplified, and then the PCR products were paired-end sequenced on an Illumina MiSeq to a depth >40,000 reads. About 99% of reads for clone 1F6 and about 100% of reads for clone 1E8 contained the variant of interest. See Supplementary Dataset 2 for crRNA and template sequences.

Off-target analysis was performed using Cas-OFFinder^43^ with the *SpCas9 from Streptococcus pyogenes: 5*ʹ*-NGG-3*ʹ model, mismatches ≤3, bulges = 0 and the hg19 genome.

### Whole-genome sequencing and analysis of the 2F3 HepG2 clone

PCR-free WGS libraries were prepared at The Center for Applied Genomics (TCAG) with Illumina’s Nextera DNA Flex Library Prep Kit, using genomic DNA from wild-type and c.1934T > G edited HepG2 cells. Both WGS libraries were sequenced to 40x mean coverage on Illumina HiSeq X. WGS alignment and variant calling was performed using BWA-MEM v0.7.12^44^ and a custom version of the human reference genome (b37) extended with an extra chromosome containing the sequence of the pEGFP-puro plasmid used for co-selection of transformants. Variant calling was performed using GATKv3.7^45^. Reads aligned to the plasmid or within 2 kb of its integration breakpoints on chr13 were collected, adapter- and quality-trimmed using Trim_Galore v.0.4.4, and de-novo assembled using SPAdes v3.9.0^46^ with k-mer sizes of 21, 33, 55, 77, 99. As independent validation of the plasmid integration, gDNA primers were designed to PCR amplify and Sanger sequence fragments spanning the putative breakpoints and a 20 nt region with no WGS coverage in the middle of the plasmid sequence. See Supplementary Dataset 1 for primer sequences.

### Edited HepG2 qPCR

Twelve replicates of wild-type, 2F3, 1F6, and 1E8 edited HepG2 cells were seeded into 12-well plates (3.5 × 10^5^ cells per well). Total RNA was extracted from the cells using the RNeasy mini-kit with Qiacube automated extraction (Qiagen). DNase was performed on-column with RNase-free DNase kit (Qiagen). RNA concentration and quality were determined by Bioanalyzer (Agilent). All RNA samples had RNA integrity numbers (RIN) above 9. First-strand synthesis was performed with 1 µg of RNA using the High-Capacity cDNA kit with random primers (Applied Biosystems). For qPCR, primers were designed to amplify *ATP7B* transcripts containing exons 5, 6, and 7 by spanning exon junctions (P2065, P2066). TBP primers were used for normalization (P378, P379). qPCR was performed with Quantstudio 5 instrument (Thermofisher) with SYBR green reagents (Thermofisher). Cycle thresholds were determined by QuantStudio software, which were used to calculate relative quantity (RQ) of *ATP7B* transcripts by relative standard curve method. See Supplementary Dataset 1 for primer sequences.

### Edited HepG2 PCR

Endpoint RT-PCR was performed on total RNA. First-strand synthesis was performed previously on RNA extracted from edited lines for qPCR (see above). The same cDNA was amplified for 30 cycles using primer pairs P243 and P244 (see Supplementary Dataset 1) to amplify cDNA with/without exon 6. PCR products were separated on 2% agarose gels stained with 0.05 % Redsafe (FroggaBio). Gel bands in the same figure were derived from the same experiment and processed in parallel.

### Edited HepG2 western blot

Total protein was isolated from wild-type, 2A1, 2F3, 1E8, and 1F6 edited HepG2 cells. Cells were first rinsed with 1x DPBS (Dulbecco’s phosphate-buffered saline, Gibco) and lysed with ice cold RIPA Buffer (Thermofisher) containing HALT protease inhibitors (Thermofisher). Cell lysate was chilled on ice for 10 min and centrifuged at 16,000 x *g* to pellet and remove cell debris. Protein amounts were determined by BCA assay (Pierce). Samples were heated at 70 °C with NuPAGE sample buffer containing NuPAGE sample reducing agent (Thermofisher). 20 µg of total protein was separated on a 10% TRIS-BIS (NuPAGE) gel at 200 V for 1 h. Separated proteins were transferred to PVDF membrane at 350 mA constant for 90 min using the Mini-TransBlot system (Bio-Rad). Transfer was validated by ponceau stain (Sigma). Membranes were blocked in 5% Milk TBST for 1 h at room temperature. PVDF membrane was cut at 75 kDa according to protein ladder (BlueElf, FroggaBio). The higher molecular weight portion of the membrane was incubated with ATP7B antibody (Abcam, Ab131208) (1/500 in 5% Milk TBST) and the lower molecular weight portion of the membrane was incubated with beta-actin antibody as a loading control (Abcam Ab8227) (1/1000 in 5% Milk TBST) overnight at 4 °C. Samples were washed three times for 10 min in TBST, and then incubated in secondary antibody (70745 anti-rabbit HRP conjugated, 1/5000 in 5% Milk TBST) (Cell Signalling) at room temperature for 1 h. The membranes were washed three times for 10 min in TBST. The images were taken after a 30 s exposure for beta-actin and after a 1 min exposure for ATP7B using the Amersham Imager 680 using Amersham ECL reagents (GE). Two replicates were obtained by repeating the initial protein isolation step. Blots in the same figure were derived from the same experiment and processed in parallel.

### Oxidative stress measurement in copper-treated HepG2 cells

WT and 2F3 HepG2 cells were initially plated at 50k cells/well and left in DMEM media (containing 10% serum and approximate copper concentration of 1.54 µM) for 3 days to become confluent. Cells were then treated for 34 h with 100–700 µM CuCl by mixing one single CuCl dose into the media, with four replicate wells for each CuCl dose. CellROX Green reagent (ThermoFisher) was diluted 1:100 in DPBS−/− to obtain a 10x dilution and then 10 µl was added to each well. One plate was prepared with WT and 2F3 HepG2 cells treated with different CuCl levels and CellROX Green; to model background signal, an additional plate was prepared with cell-free media that was kept in the incubator and treated as the first plate. The two plates were read 14 h after adding CellROX Green (corresponding to 34 h after adding CuCl), using a BioTek neo2 plate reader with a bottom read 3 × 3 area scan, excitation of 485(20)/emission of 520(10) and 100 gain. The fluorescence readout corresponding to each CuCl dose in the first plate was background-corrected by subtracting the average background signal of the corresponding four wells in the cell-free plate.

### Preprints

An earlier version of this manuscript has been published on bioRxiv^47^.

### Reporting summary

Further information on experimental design is available in the Nature Research Reporting Summary linked to this paper.

## Supplementary information

Supplementary Information

Reporting Summary Checklist

Supplementary Data 1

Supplementary Data 2

## Data Availability

All relevant data are provided in the supplementary datasets or are publicly available. 2F3 HepG2 whole-genome sequencing data are available from SRA as accession SRX7805353 (study PRJNA608850, sample SAMN14210548). Edited HepG2 cells described in this work will be made available upon request for academic and not-for-profit research purposes only.

## Code availability

For splicing prediction, the deep-neural network model, training data, and performance evaluation data are proprietary to Deep Genomics and thus they are not made available.
