# Supplementary material for: ATP7B variant c.1934T > G p.Met645Arg causes Wilson disease by promoting exon 6 skipping
Source: NPJ Genom Med. 2020 Apr 8;5:16. doi: 10.1038/s41525-020-0123-6 (PMC7142117; doi:10.1038/s41525-020-0123-6)
Supplement: Supplementary file 1 — Supplementary Information [file 41525_2020_123_MOESM1_ESM.pdf]

Supplementary Figures

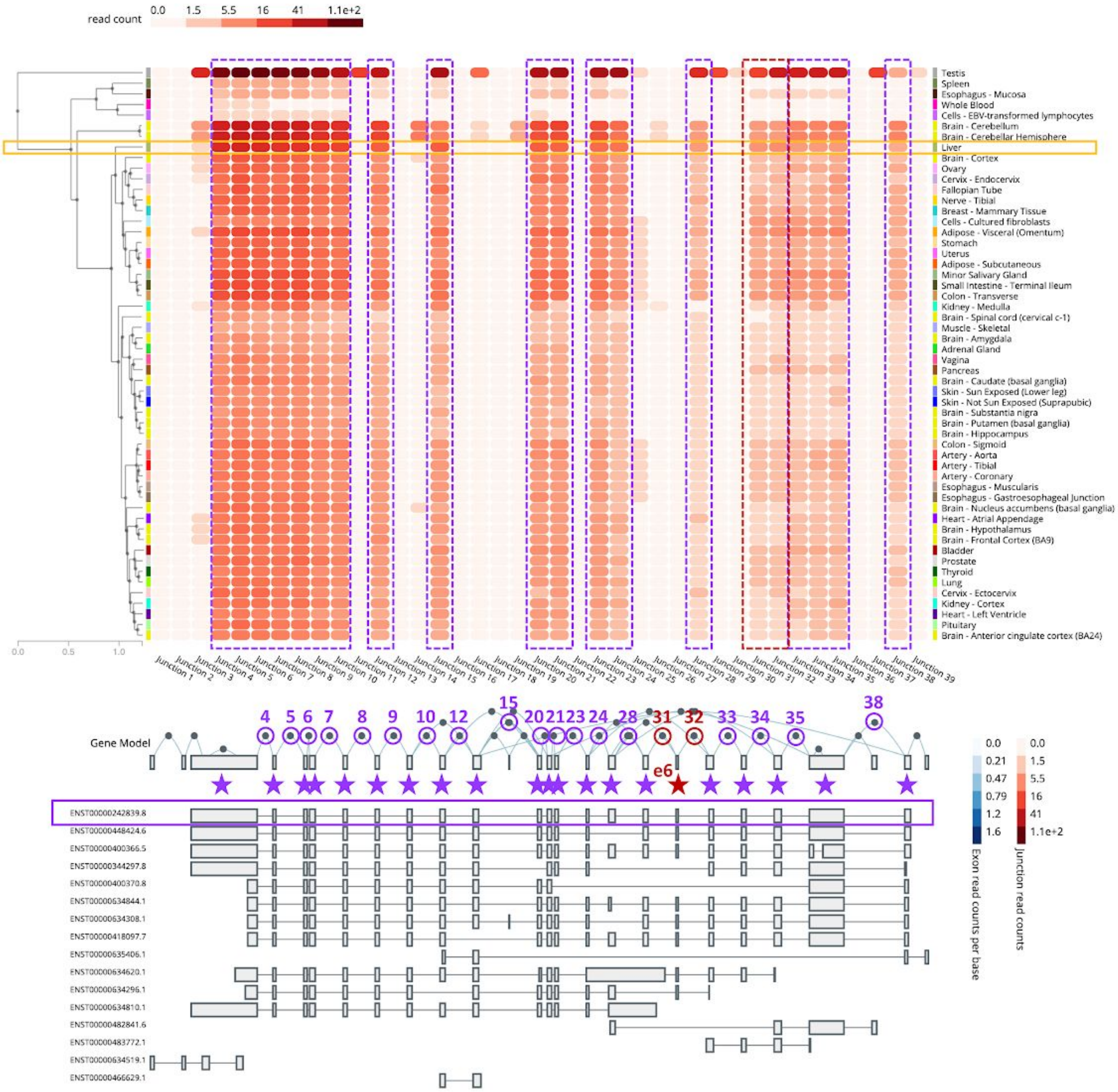

Supplementary figure 1: manual analysis of GTEx junctions suggests ENST00000242839 (highlighted by the purple box) is the highest expressed isoform in liver (highlighted by the orange box). In the gene model diagram, well-supported junctions in liver RNA-seq are highlighted by circles and numbered, so that their level of support can be looked up in the heatmap above; the corresponding exons are highlighted by stars; exon 6 (e6) and its supporting junctions (31 and 32) are highlighted in red, all other junctions and exons are highlighted in purple. Exons numbers refer to ENST00000242839. The gene is displayed with the 3’ on the left and 5’ on the right; the exon numbering increases right-to-left, whereas the junction numbering follows the opposite order.

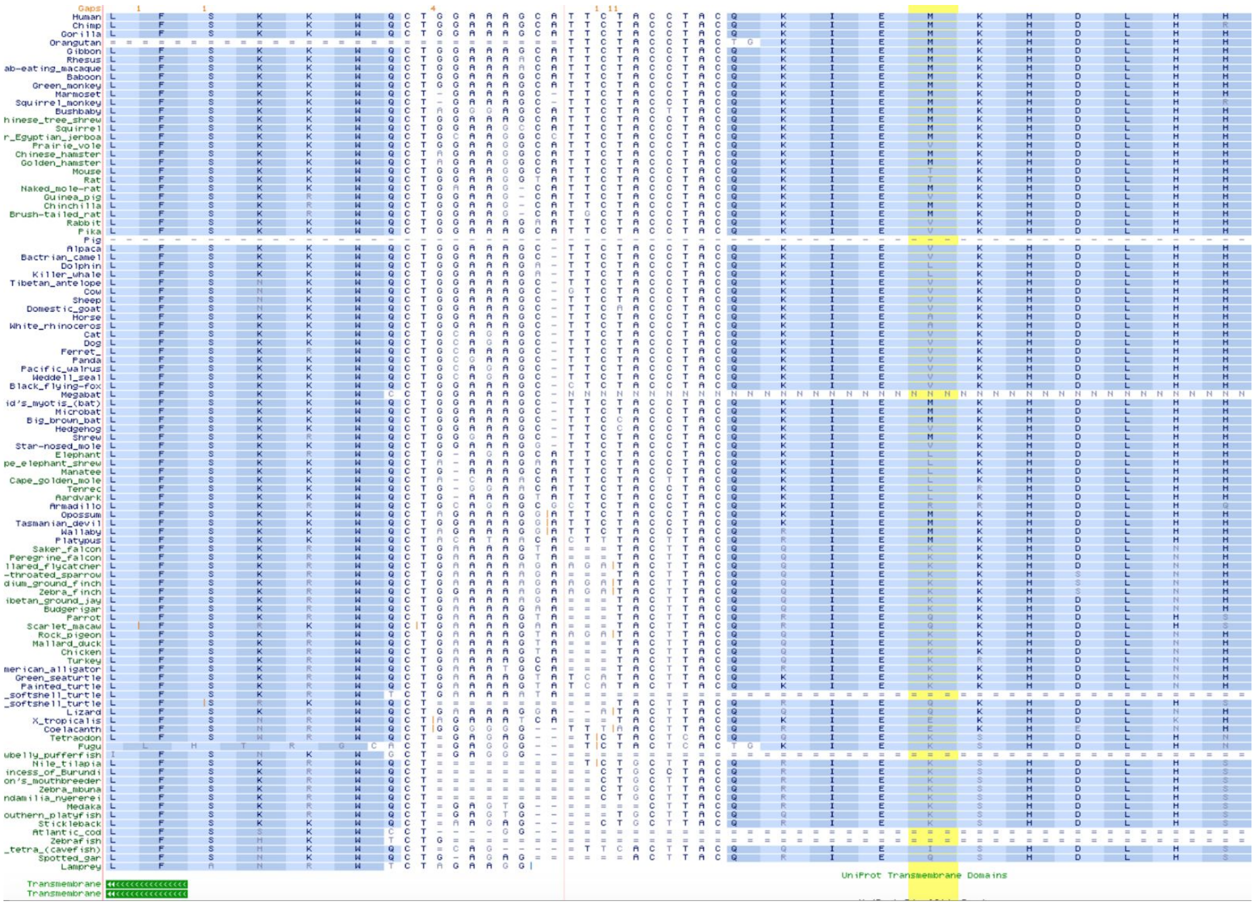

Supplementary figure 2: UCSC browser 100 vertebrate multiple sequence alignment showing amino acid substitution patterns for Met645 (exon 6 is on the right, exon 7 is on the left).

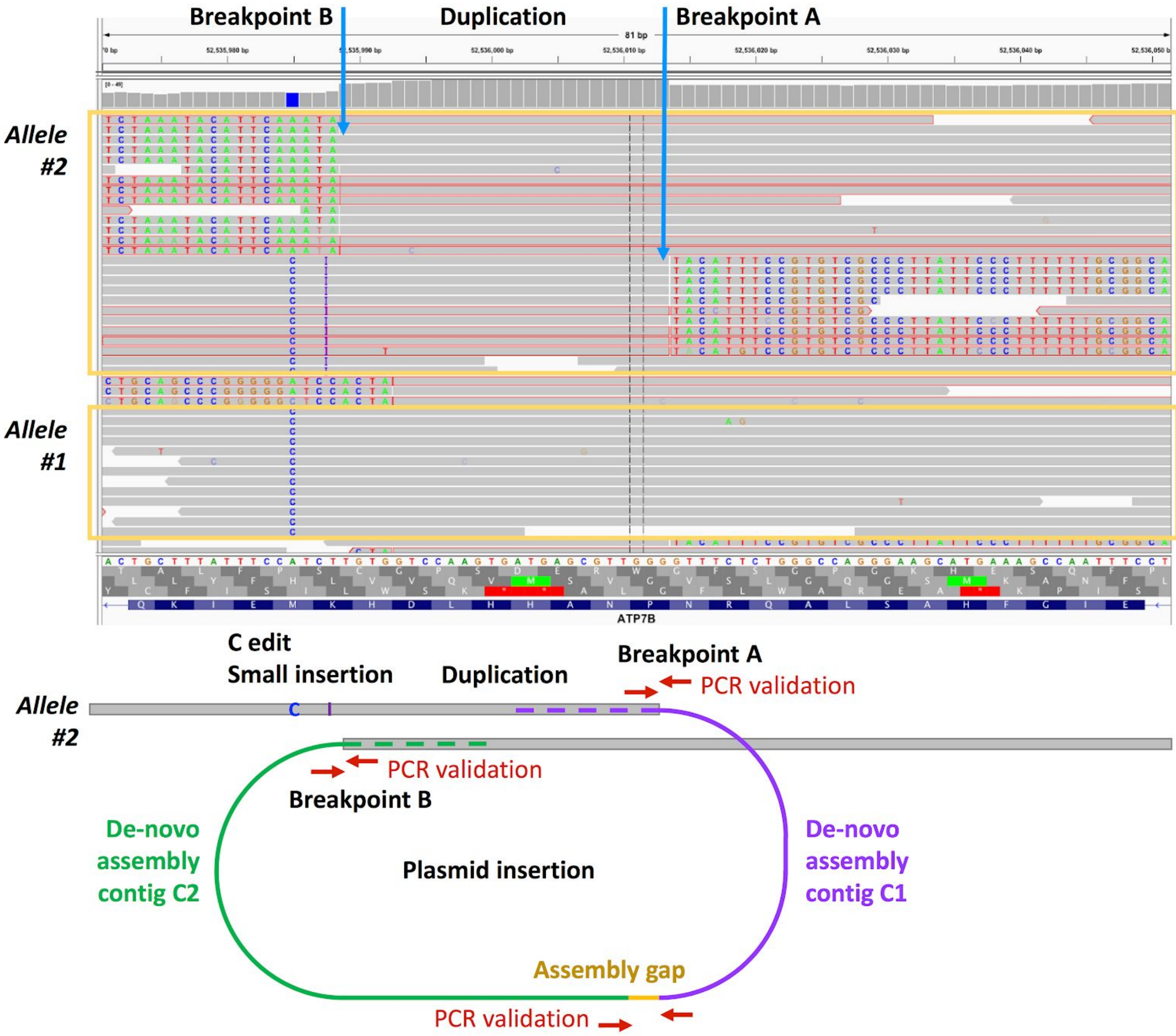

Supplementary figure 3: WGS results for the 2F3 HepG2 clone. Top: alignment to the human reference sequence, visualized using the Integrative Genomics Viewer (IGV), reveals three major read clusters, one corresponding to the edited c.1934T>G allele (allele 1) and the others suggesting a partial exon 6 duplication and plasmid insertion (allele 2). Bottom: the reconstructed sequence of allele 2, showing the human genome reference sequence as gray blocks, the contigs obtained by de-novo assembly as green and violet lines, the assembly gap as a gold line, the PCR primer sets used for validation as red arrow pairs (see Supplementary Note 1 for more details, including PCR results); note that the plasmid insertion length is not proportional to the exon 6 length and that the de-novo assembly contigs span the exon 6 as well as nearby genomic reference sequence as suggested by the dashed lines.

Clone 1E8

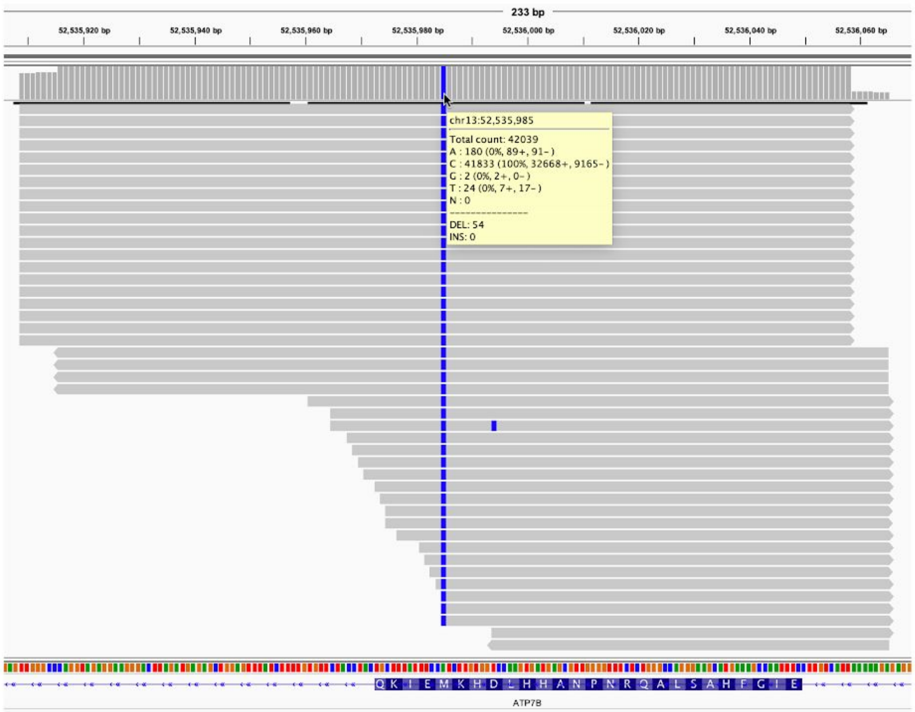

Clone 1F6

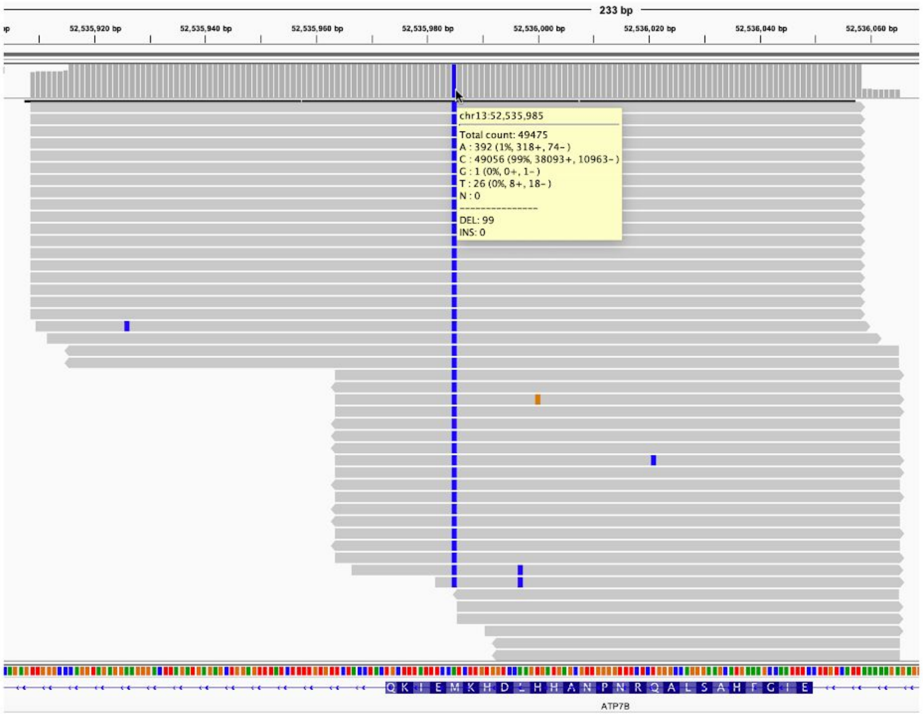

Supplementary figure 4: MiSeq amplicon sequencing of *ATP7B* exon 6 demonstrates that HepG2 clones 1E8 and 1F6 are homozygous for c.1934T>G (chr13:52535985:A>C). Reads were down-sampled and visualized in IGV; nucleotide counts reflect the full read set.

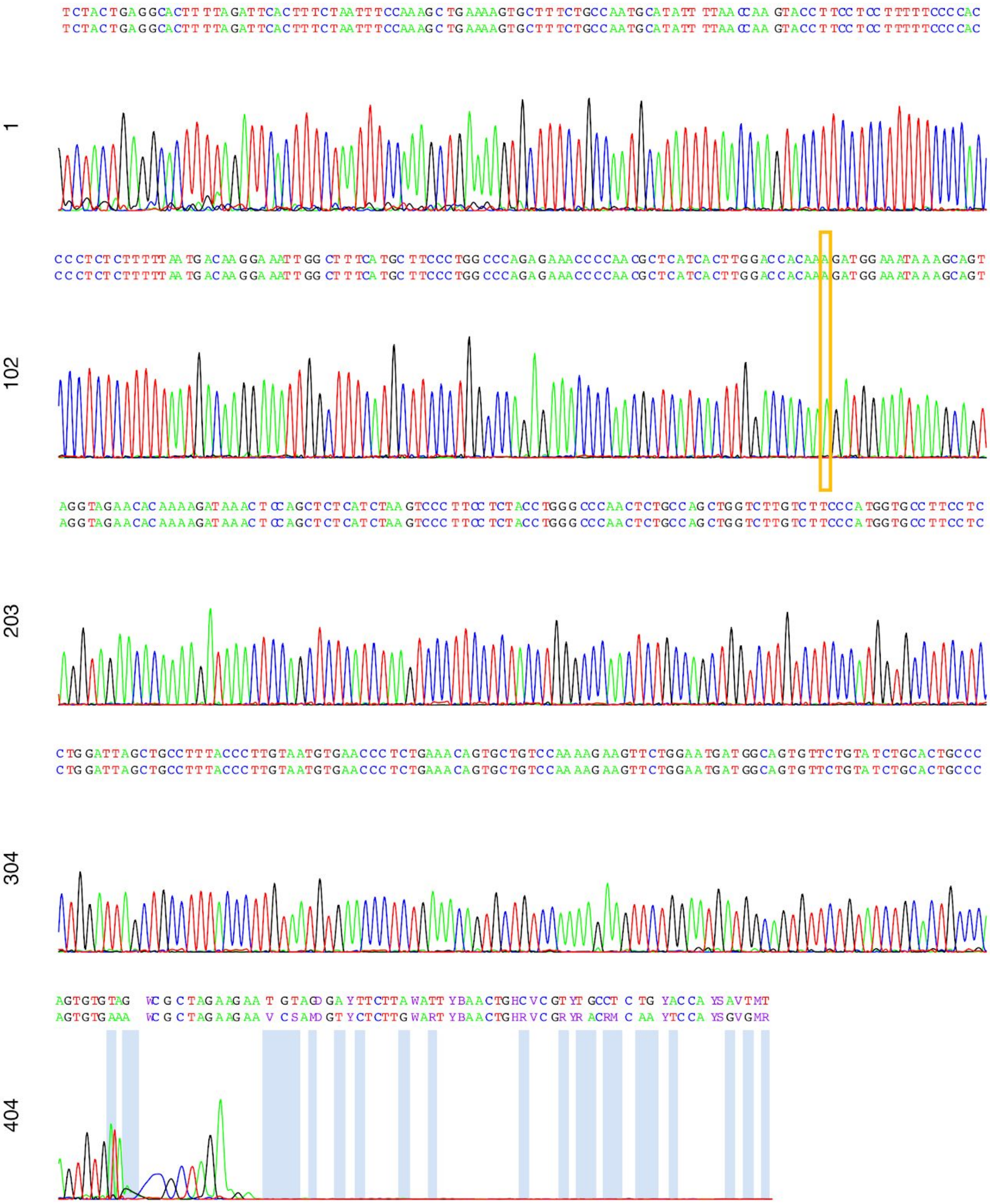

Supplementary figure 5: Sanger sequencing of *ATP7B* exon 6 demonstrates that HepG2 clone 2A1 is homozygous for c.1931dupA (chr13:52535987:C>CT). Sanger reads correspond to the negative strand and thus are in reverse complement of the human reference sequence; the A insertion is identified by an orange box.

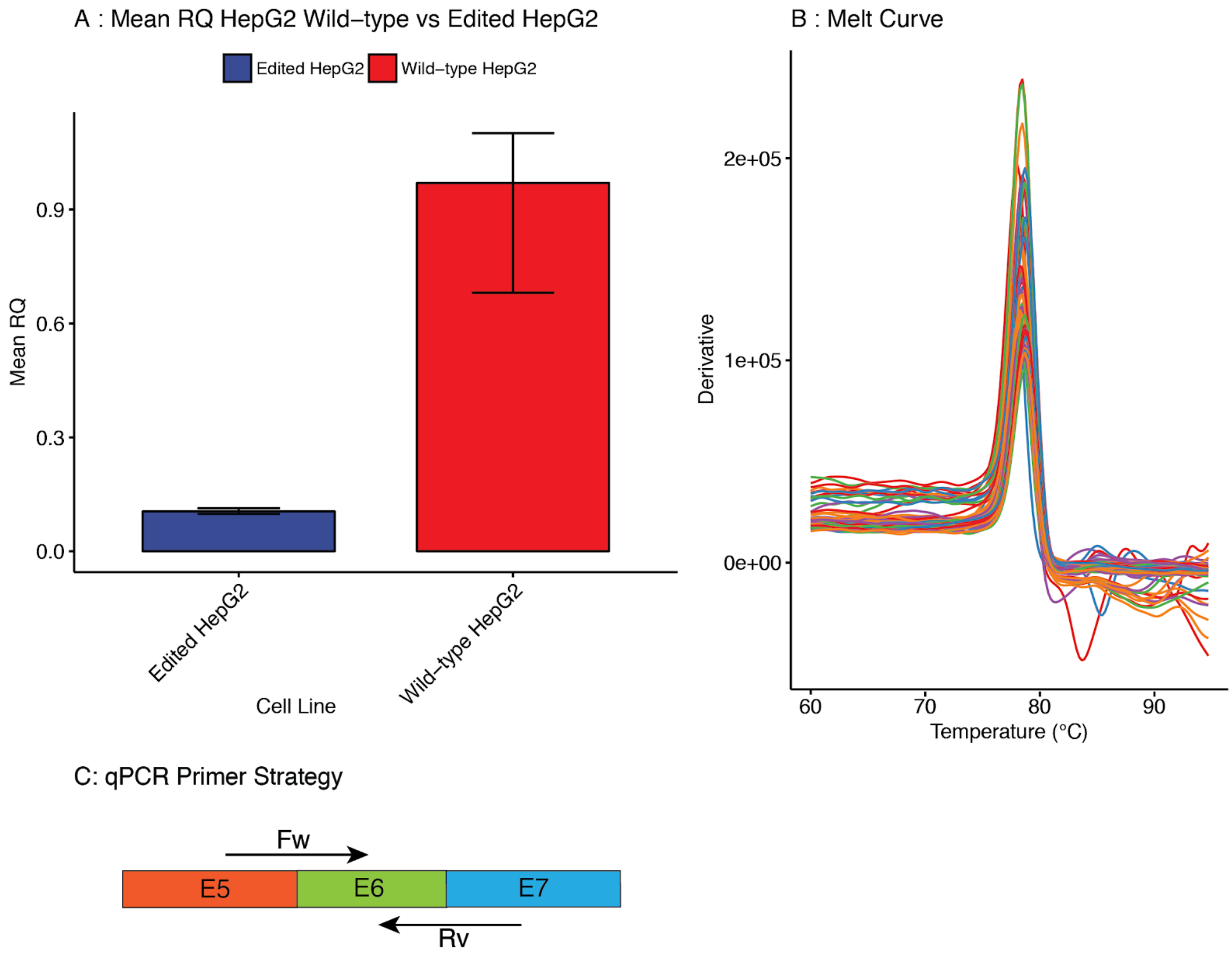

Supplementary Figure 6: qPCR for relative quantity (RQ) of transcripts containing exons 5, 6 and 7 between wild-type HepG2 and edited 2F3 cells. (A) Compared to wild-type cells, edited HepG2 cells have reduced exon 5, 6, 7 spanning transcript; the barplot displays the mean RQ of 12 independent RNA extractions for each cell line, with error bars corresponding to minimum or maximum RQ. (B) Melt curve for all biological replicates is indicative of a single PCR product. (C) PCR strategy with forward (FW) and reverse (RV) primers spanning the boundaries between exons 5 and 6 and 6 and 7 respectively.

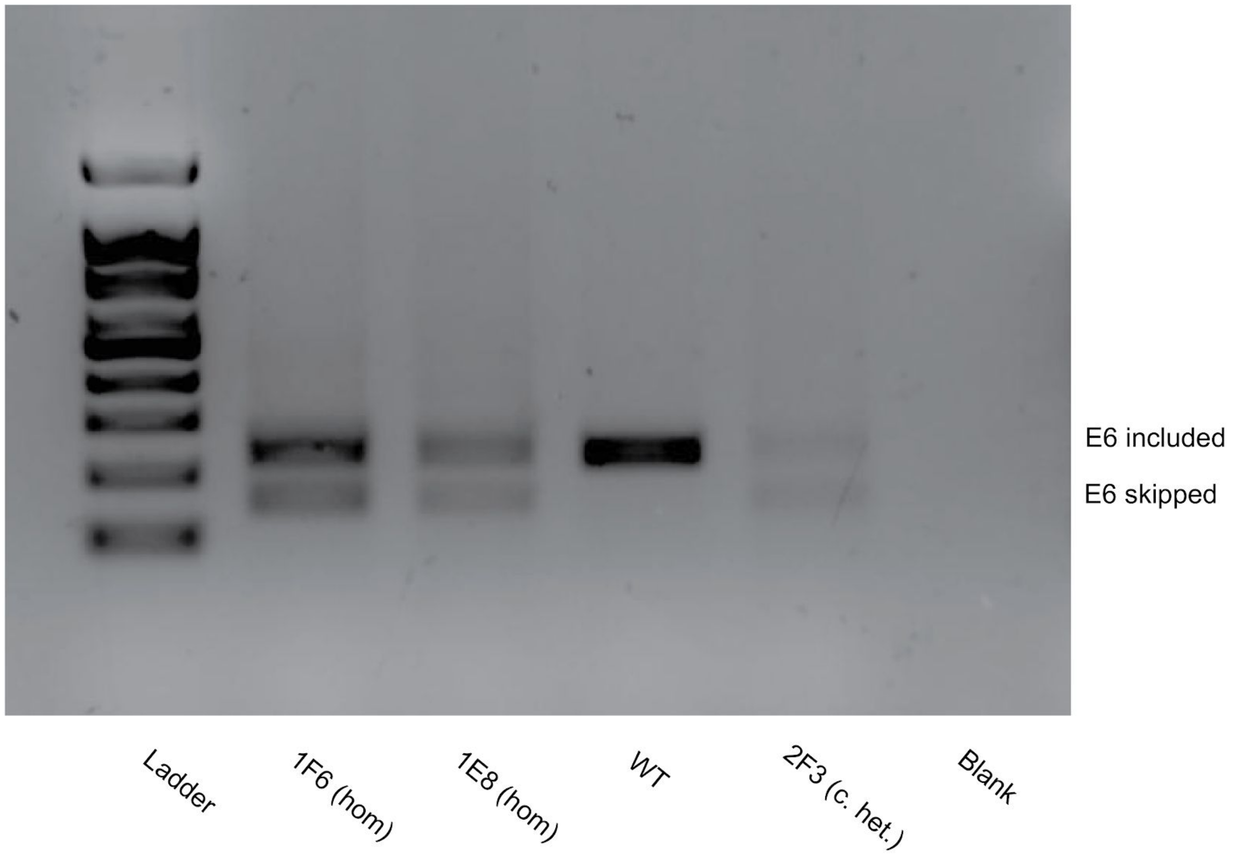

Supplementary Figure 7: PCR conducted on a single replicate each of HepG2 edited clones 1F6 (homozygous), 1E8 (homozygous) and 2F3 (compound heterozygous with a large structural rearrangement) shows partial exon 6 skipping, as expected, whereas no skipping is detected in WT HepG2. Note that NMD is expected to remove isoforms lacking exon 6 or produced by the allele with a large structural rearrangement, thus band intensity ratios are not directly reflective of percentage exon inclusion.

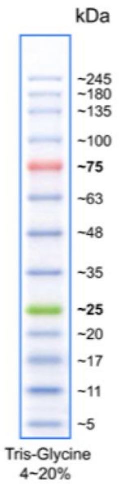

Lane	Sample
1	Ladder
2	HepG2 (wt)
3	2A1 (k.o)
4	2F3
5	1E8
6	1F6
7	Ladder
8	Ladder

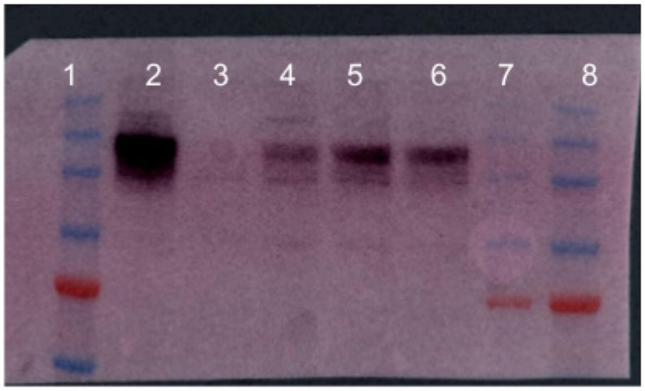

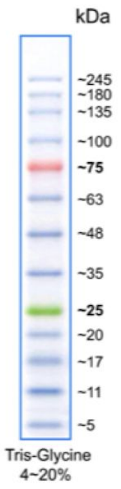

Lane	Sample
1	HepG2 (wt)
2	2A1 (k.o)
3	2F3
4	1E8
5	1F6
6	Blank
7	Blank
8	Blank
9	Blank
10	Ladder

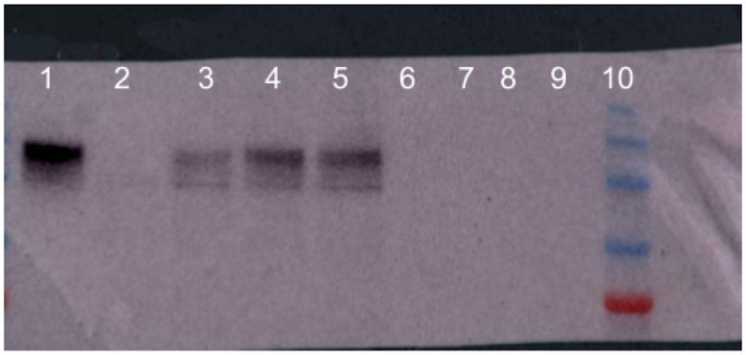

Supplementary figure 8: full gel images for the two western blot replicates, with different time exposures (top: 2 minutes; bottom: 1 minute, same as displayed in the main text Figure 3). Replicates were obtained by repeating protein isolation.

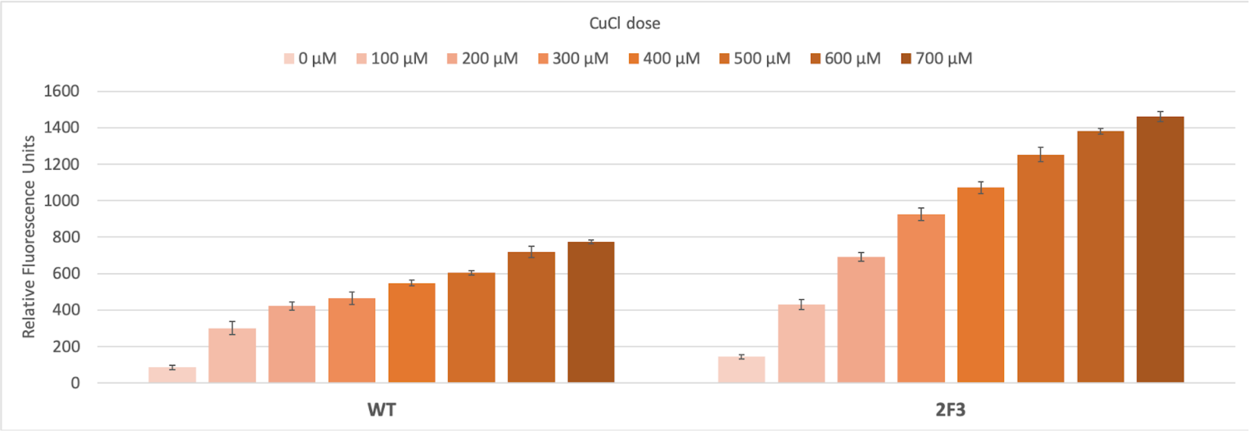

Supplementary figure 9: 2F3 HepG2 cells exhibit greater oxidative stress than WT HepG2 in response to copper challenge. Cells were treated with 0-700 µM CuCl. Reactive oxygen species were measured using fluorescence induced by CellROX Green oxidation (y axis). Error bars represent one standard deviation unit and each fluorescence measurement was performed on four replicate wells.

Supplementary Note 1

(1) ATP7B Isoforms **2**

(2) In-silico predictions specificity **9**

(3) 2F3 HepG2 Clone Characterization by WGS **10**

(4) Frequency and Prevalence Calculations **14**

References **16**

(1) ATP7B Isoforms

NCBI Homo sapiens RefSeq annotation release 109 has five curated isoforms: NM_000053 (21 exons, encoding 1465 amino acids), NM_001243182 (22 exons, encoding 1354 amino acids), NM_001330578 (20 exons, encoding 1387 amino acids), NM_001330579 (19 exons, encoding 1381 amino acids), NM_001005918 (17 exons, encoding 1258 amino acids).
In the ClinVar data downloaded in January 2019, NM_000053 is used for 209 / 210 submissions of pathogenic or likely pathogenic variants.
Accordingly, based on annotation, protein domain composition and conservation evidence, NM_000053 is categorized as principal by APPRIS 2019_02.v29 [Rodriguez 2013]. In contrast, NM_001243182, NM_001330578 and NM_001330579 are categorized as alternative, whereas NM_001005918 is categorized as minor.
Manual review of junctional counts from GTEx v8 [GTEx 2013] reveals that ENST00000242839 comprises the best supported junctions (see Figure 1.1). ENST00000242839 is almost identical to NM_000053, since they differ by only a few nucleotides at the 3'UTR exon 21 end.
An isoform lacking only exon 6 is not expected to be stable, as it would be liable to NMD-mediated degradation and, in any case, the mutant protein would present a shift of the majority of its reading frame and it would probably misfold and undergo degradation. GTEx does not suggest the presence of an isoform lacking only exon 6. Similarly, RT-PCR in HepG2 cells using primers on exon 5 and exon 7 shows no evidence of exon 6 skipping (see Figure 1.2).
A shorter isoform lacking exons 6, 7, 8 and 12 (corresponding to NM_001005918 and ENST00000344297) has been described in the literature as expressed in brain but not in liver [Petrukhin 1994]. Inspecting GTEx junctions, this isoform appears to be present at relatively low levels in cerebellum, but not in liver or other brain regions (see Figure 1.3). This shorter isoform lacks two transmembrane domains and localizes predominantly to the cytoplasm rather than to the Golgi [Yang et al. 1997]. The functional role of this isoform is unclear; its incapability to localize to the Golgi and its absence in hepatocytes suggest that it cannot replace the main isoform in regulating copper levels by promoting copper excretion into the bile and ceruloplasmin copper loading.
Two additional isoforms are even shorter and do not include exon 6. ENST00000400370 skips exons 3-10, but the corresponding junction is not expressed in any GTEx tissue or cell type. ENST00000635406 contains only four exons, and its junctions are not expressed either (see Figure 1.4). Other isoforms lacking exon 6 contain few exons or lack many exons from the 5' portion and were not reviewed in detail.
Incidentally, cerebellum (but not other brain regions) presents an additional alternate splicing event, the inclusion of a micro-exon of 20 bp between exon 12 and 13 (exon numbering based on ENST00000242839). The inclusion of this exon must be combined with alternative splicing of at least another exon for the resulting transcript to be in-frame (and presumably stable). ENST00000634308 presents the inclusion of this micro-exon and the exclusion of exon 8, but the junction reads supporting exon 8 skipping are not present in cerebellum (see Figure 1.5).

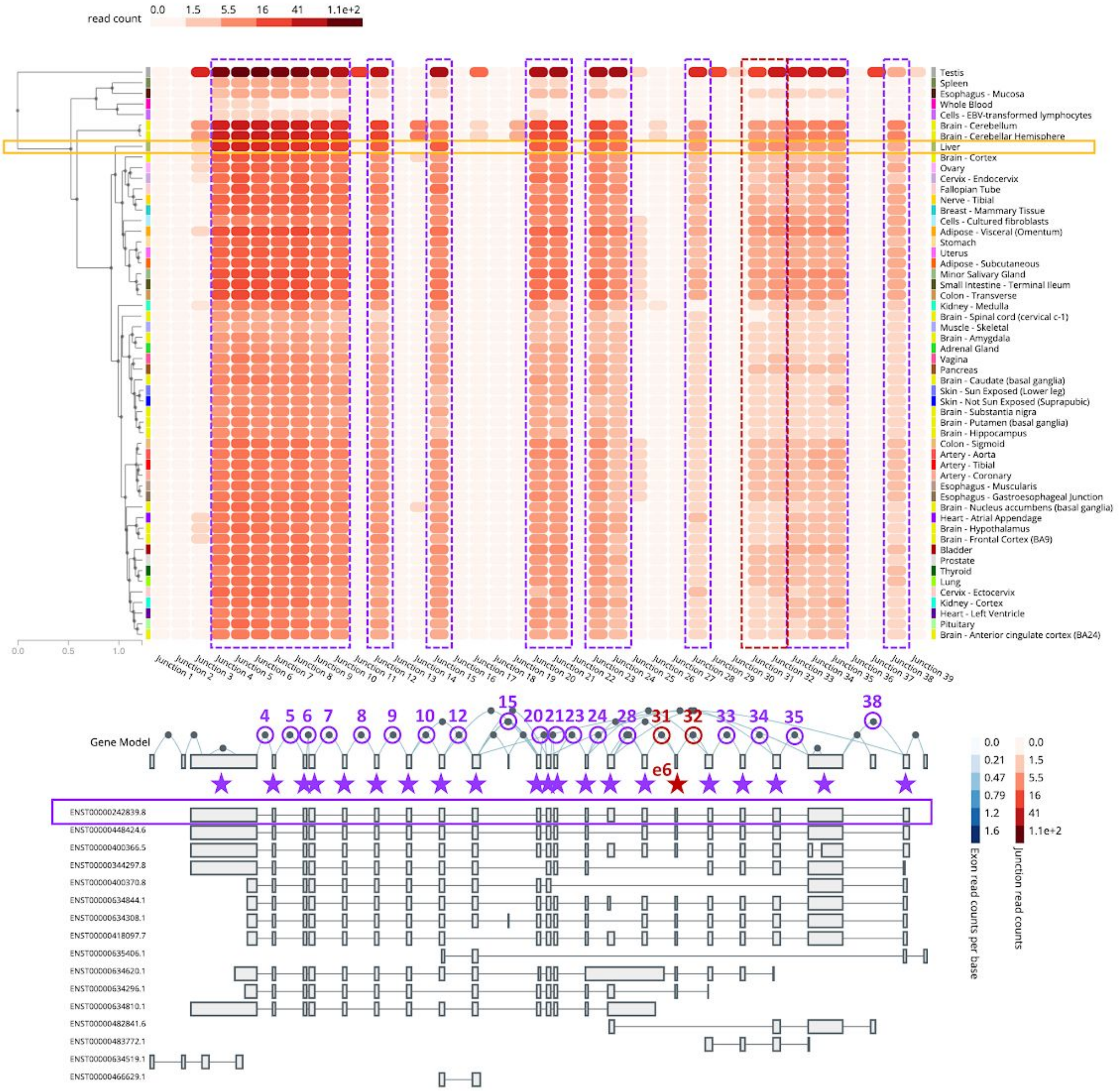

Figure 1.1 (same as supplementary figure 1): manual analysis of GTEx junctions suggests ENST00000242839 (highlighted by the purple box) is the highest expressed isoform in liver (highlighted by the orange box). In the gene model diagram, well-supported junctions in liver RNA-seq are highlighted by circles and numbered, so that their level of support can be looked up in the heatmap above; the corresponding exons are highlighted by stars; exon 6 (e6) and its supporting junctions (31 and 32) are highlighted in red, all other junctions and exons are highlighted in purple. Exons numbers refer to ENST00000242839. The gene is displayed with the 3' on the left and 5' on the right; the exon numbering increases right-to-left, whereas the junction numbering follows the opposite order.

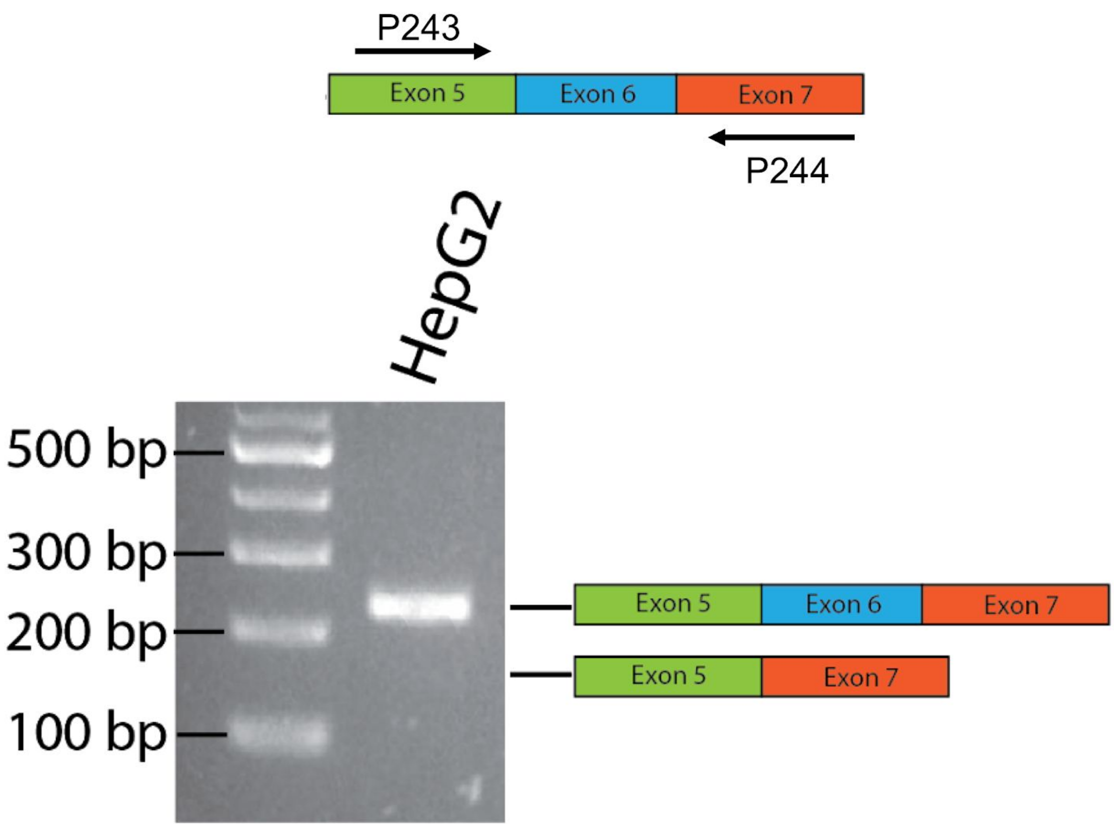

Figure 1.2: total RNA from WT HepG2 cells suggests the absence of isoforms lacking only exon 6. RNA was extracted using Qiagen RNeasy kit and reverse transcribed using high-capacity cDNA kit with random primers; PCR was performed with primers P243 (CCAGCAAAGCCCTTGTTAAG) and P244 (GCTCGTTGCTGGGTATCAG), which overlap exon 5 and exon 7 respectively.

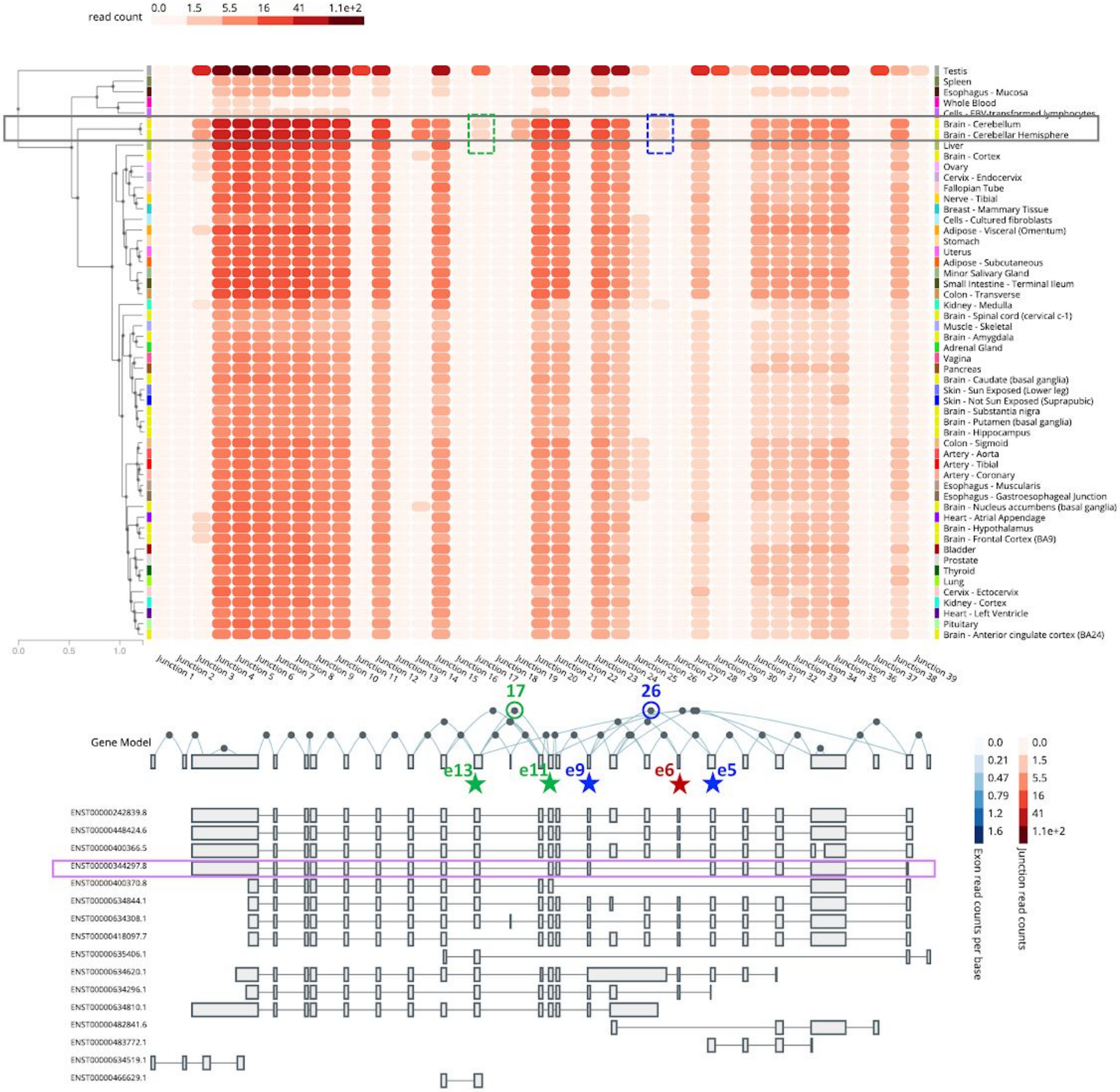

Figure 1.3: manual analysis of GTEx junctions suggests the presence of a minor isoform (ENST00000344297, highlighted by a pink box), expressed at relatively low levels in brain but not in liver, lacking exons 6-8 and exon 12; the supporting junctions, 17 and 26, are highlighted in green and blue respectively. Note that exons are numbered relative to ENST00000242839.

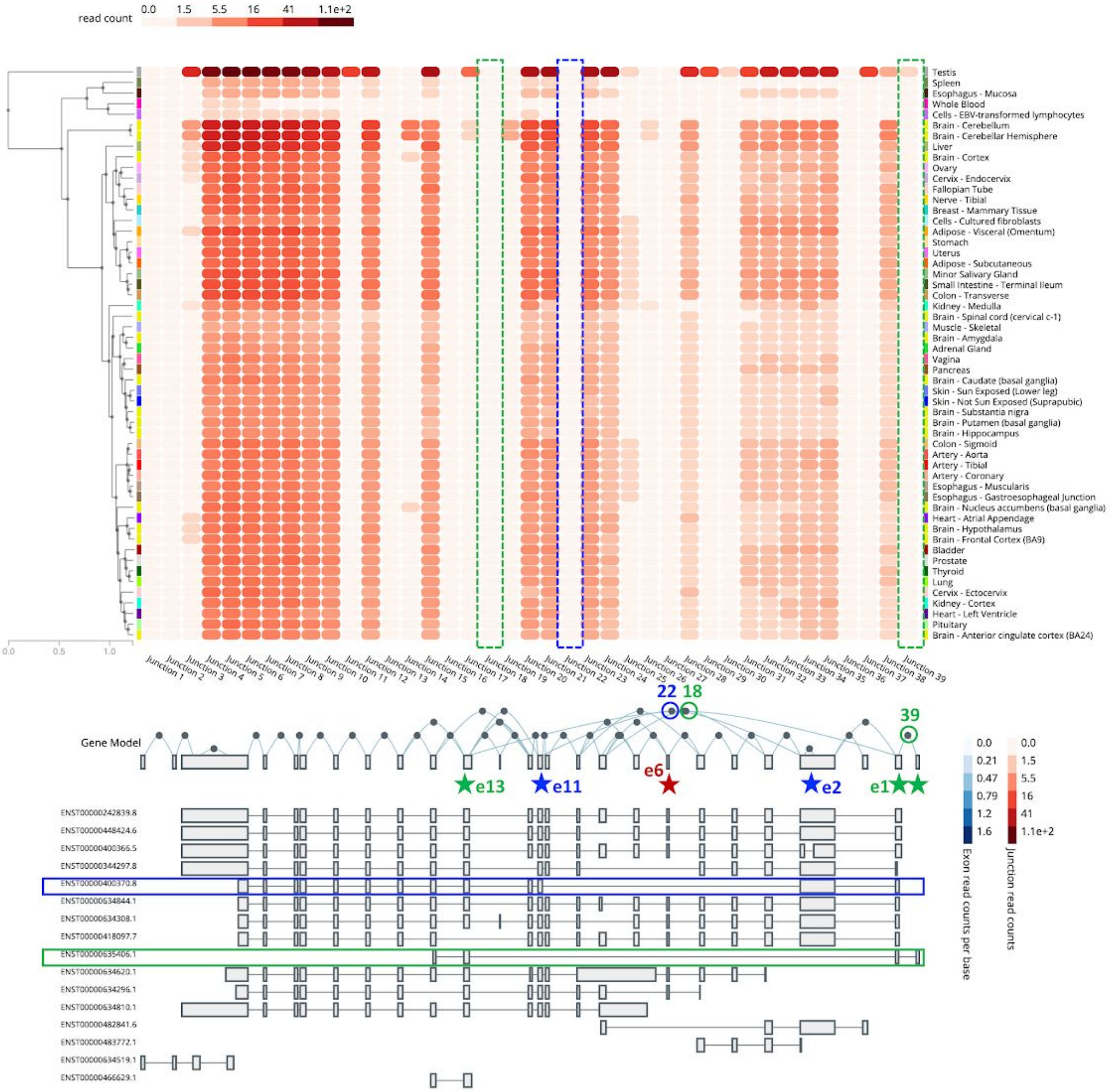

Figure 1.4: ENST00000400370 lacks exons 3-10 and the junction supporting it (junction 22) has very low read count in GTEx, suggesting this isoform is expressed at very low levels or not expressed; this isoform and junctions are highlighted in blue. ENST00000635406 contains only four exons, and the junctions supporting it (18 and 39) have very low read count in GTEx; this isoform and junctions are highlighted in green.

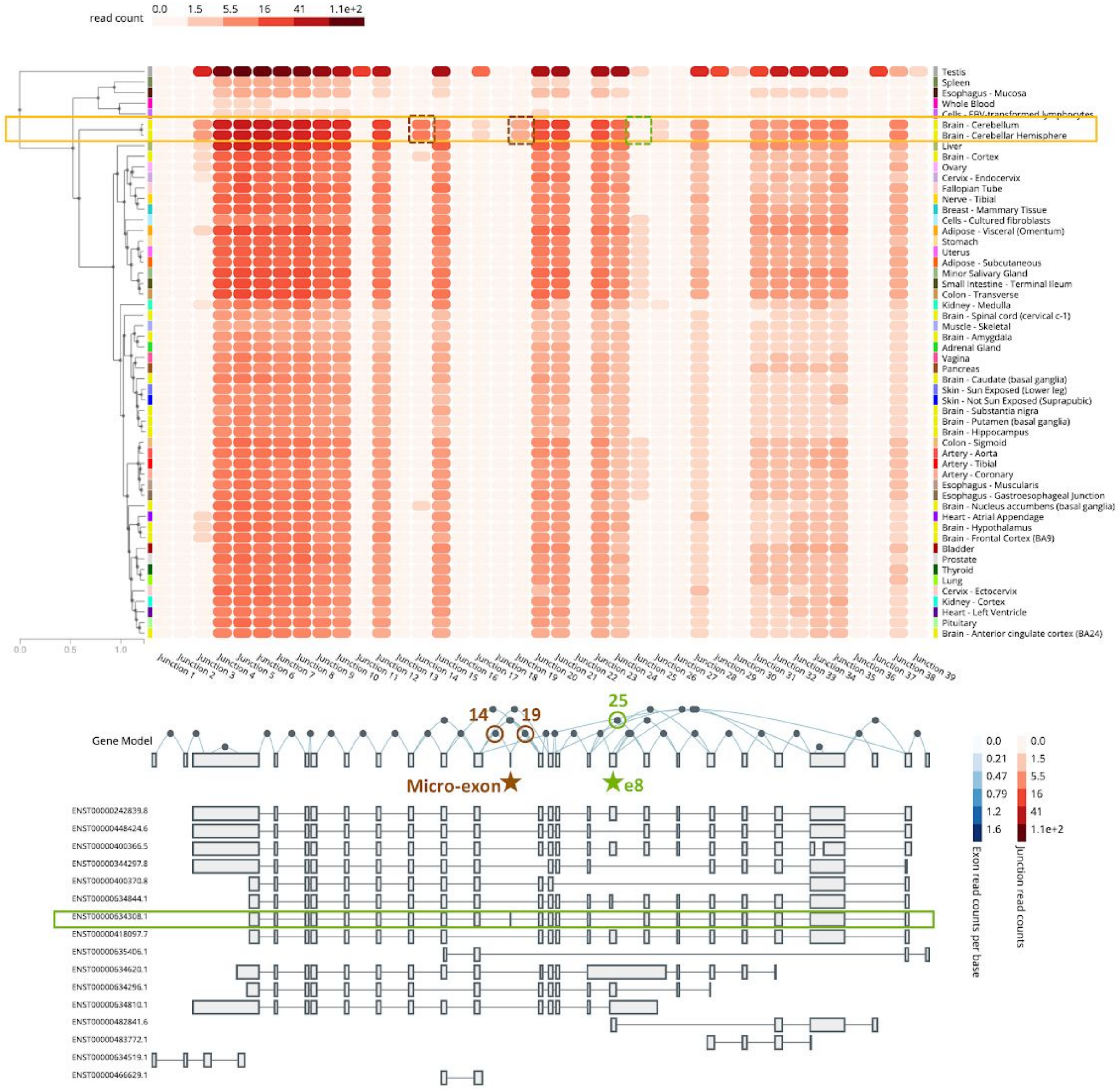

Figure 1.5: cerebellum also presents the inclusion of a 20 bp micro-exon located between ENST00000242839 exon 12 and 13 (supporting junctions and exon highlighted in brown). ENST00000634308 presents the inclusion of this micro-exon and the exclusion of exon 8, but the junctions supporting the exclusion of exon 8 are not expressed (supporting junctions and exon highlighted in green).

It is also noteworthy that western blot showed only one band in WT and edited HepG2 cells (see main text Figure 3 and Supplementary Figure 7). According to the manufacturer[1], the antibody we used recognizes the protein region comprised between amino acid 150 and 250, which is

[1] https://www.abcam.com/atp7b-antibody-epr6793-ab131208.html

located in exon 2 of NM_000053, thus we expect that it should be able to recognize the shorter protein isoform lacking exons 6-8 and 12 if it were present.

In conclusion, multiple lines of evidence support using NM_000053 (or ENST00000242839) as the principal transcript for ATP7B in hepatocytes.

(2) In-silico Predictions Specificity

Using our splicing predictor (DSN), a large delta score corresponds to a large change in exon recognition, resulting in exon skipping or sometimes in alternative splice site usage. As a reference:

- When considering the splicing consensus sequence (acceptor: 3 bp exonic and 20 bp intronic around the splice site; donor: 3 bp exonic and 6 bp exonic around the splice site; while excluding the highly conserved GT/AG dinucleotide, for both donor and acceptor), and classifying ClinVar pathogenic or likely pathogenic variants versus benign or likely benign variants, a delta score < -0.9621 corresponds to a false positive rate of 5% and a true positive rate of 93%
- When considering the MaPSy mini-gene assay variants, and classifying variants with skipping > 50% versus variants with limited or no skipping, a delta score < -0.3871 corresponds to a false positive rate of 5% and a true positive rate of 65%

The score for c.1934T>G Met645Arg (chr13:52535985:A:C) is -0.7337561, which suggests skipping > 50%, as discussed in the main text of the manuscript.

To demonstrate specificity of DSN splicing predictions, we downloaded all ATP7B variants from gnomAD v2.1.1, excluding c.1934T>G Met645Arg (N = 2,152), and ran the predictor. We then calculated the max allele frequency based on the African, European non-Finnish, Latin American, East Asian and South Asian gnomAD populations (pooled over exomes and genomes), and grouped variants in three bins:

- Variants with max allele frequency (0.005,1] were deemed very unlikely to be pathogenic, because mere homozygosity for one of these variants would result in a disorder up to 0.005^2 = 2.5 / 100,000, where the Wilson Disease prevalence is broadly reported as 3.3 / 100,000
- Variants with max allele frequency (0.001,0.005], like c.1934T>G Met645Arg, were considered potentially pathogenic, but some might have a mild effect and be pathogenic only in combination with other variants.
- Variants with frequency ≤ 0.001 were considered potentially pathogenic

Therefore, we expected that very few or no variants in the first group would exceed the c.1934T>G Met645Arg DSN delta score, and that the third group should be the most enriched in variants with large splicing effect predictions. This is exactly what we observed (see Table 2.1, Figure 2.1, Figure 2.2).

Max frequency bin	Number and percentage variants with DSN delta < c.1934T>G Met645Arg DSN delta
[0, 0.001]	174/2050 (8%)

(0.001, 0.005]	3/53 (6%)
(0.005, 1]	0/49 (0%)

Table 2.1: number and percentage of ATP7B variants receiving a DSN delta score indicating a larger splicing alteration than c.1934T>G Met645Arg, stratified by maximum frequency bin. Variants with maximum frequency in the (0.005, 1] bin are not expected to be pathogenic.

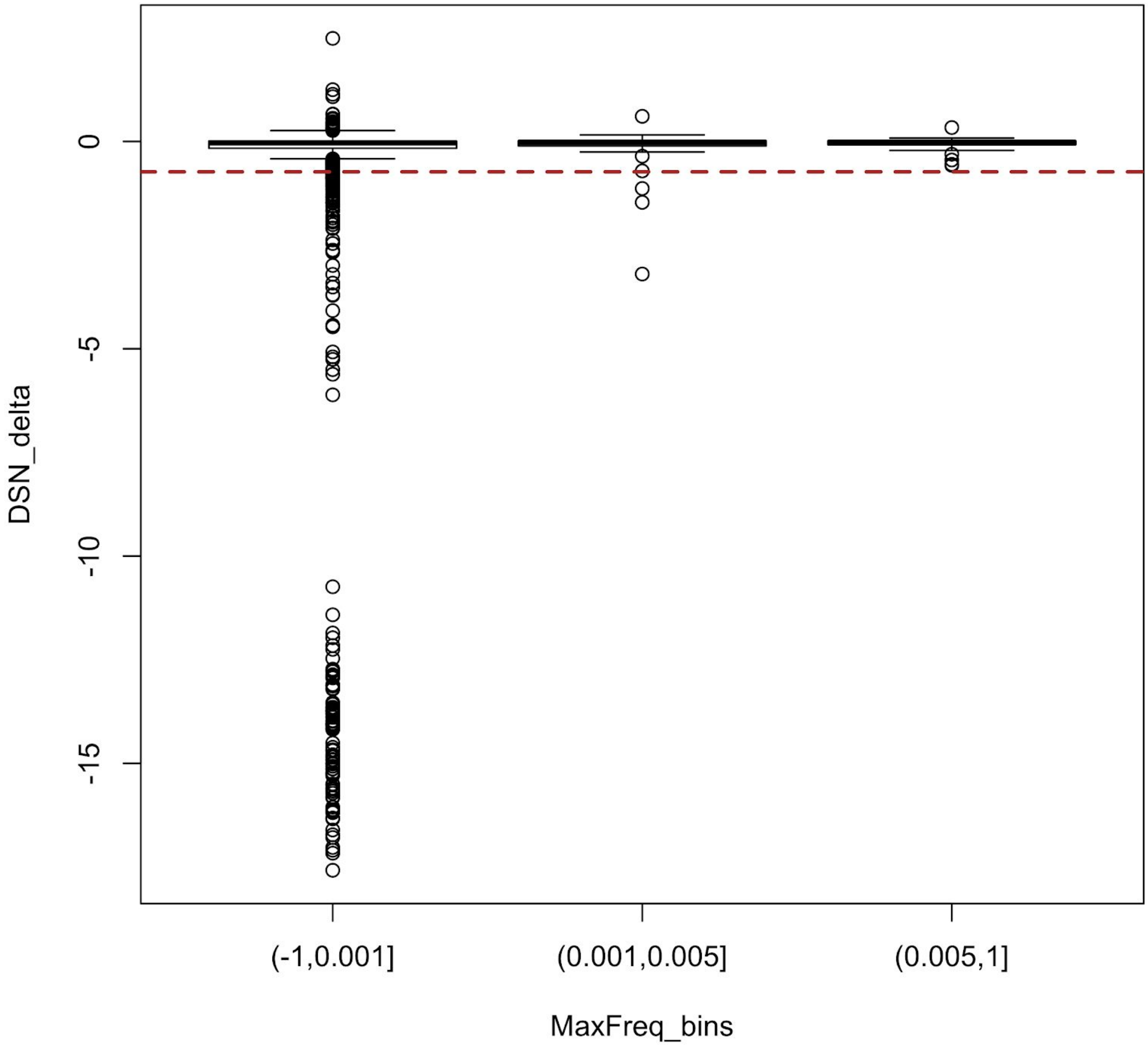

Figure 2.1: boxplot of DNS delta splicing predictions, for each ATP7B variant group. Variants with maximum frequency in the (0.005, 1] bin are not expected to be pathogenic and have predictions not exceeding the c.1934T>G Met645Arg value (-0.7337561, red dotted line).

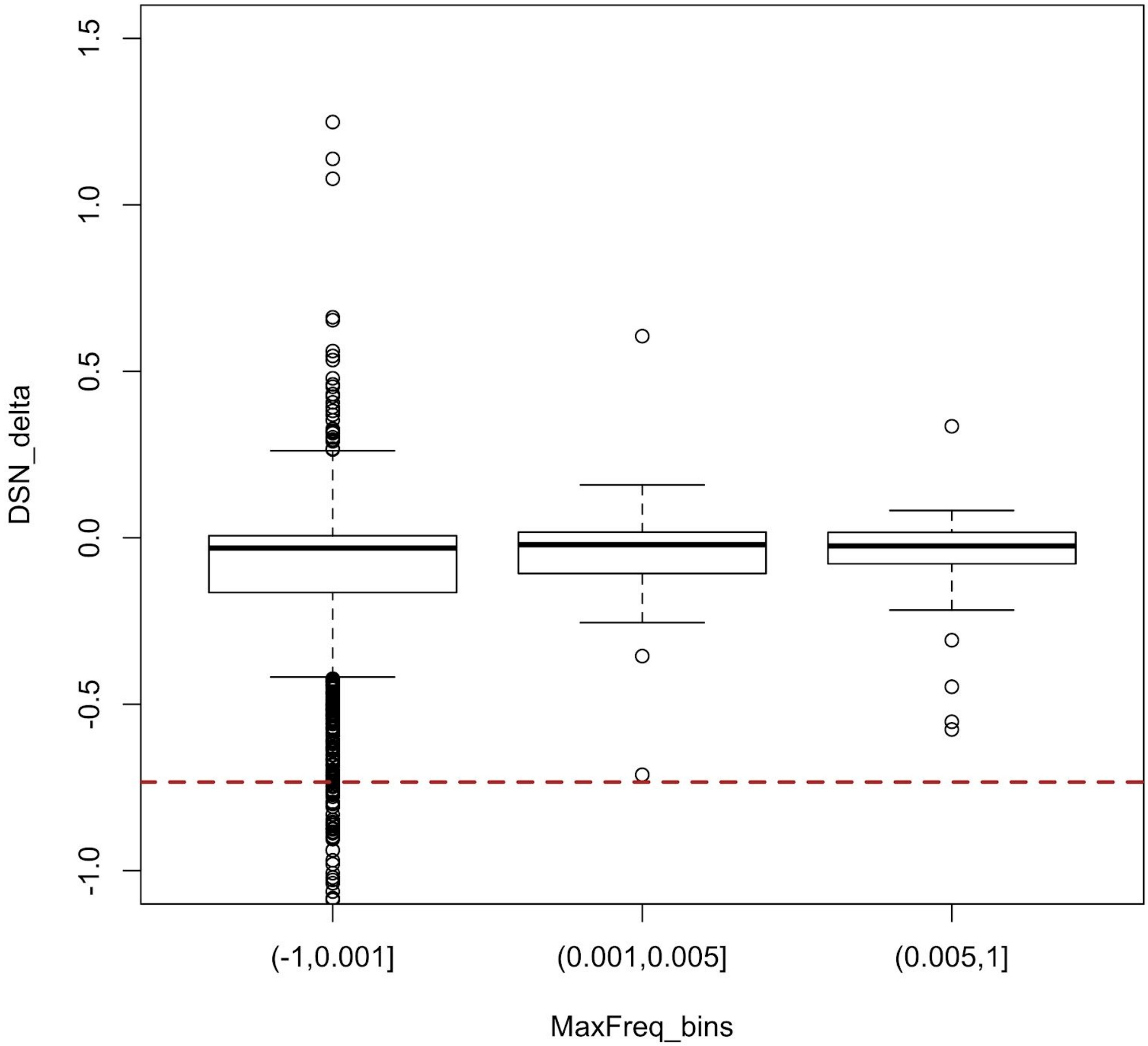

Figure 2.2: the same plot as in Figure 2.1, zoomed to a smaller DSN delta score region, to highlight that the two largest negative scores for the (0.005, 1] bin not exceeding the c.1934T>G Met645Arg value (-0.7337561, red dotted line).

(3) 2F3 HepG2 Clone Characterization by WGS

WGS alignment to the human reference sequence revealed three major read clusters at the ATP7B exon 6 locus. One corresponded to the c.1934T>G allele, showing no other edits or alterations. The other two clusters, corresponding to the second allele, had one portion of the reads aligning to the reference sequence, whereas the other portion of the reads was completely different than the human reference sequence and instead corresponded to a section of the co-transfection plasmid (see Figure 3.1 for the alignment results and Figure 3.2 for the plasmid structure). Based on these results, we inferred the presence of a partial exon 6 duplication flanking a plasmid sequence insertion; we used genomic PCR to validate the breakpoints joining human reference sequence to plasmid sequence (see Figure 3.1 for the diagram describing the reconstructed allele). To resolve the full length of the plasmid insertion, we aligned all reads to the human reference extended by the plasmid sequence as an additional chromosome, and retrieved all reads mapping to the plasmid and to 2 kb of reference sequence around the breakpoints; we then performed de-novo assembly and identified two contigs, which included the human-plasmid breakpoints but were separated by a small assembly gap when compared to the original plasmid sequence (see see Figure 3.1 for the de-novo assembly results). We hypothesized that the small assembly gap was due to a region with low sequencing coverage, and we confirmed this by genomic PCR spanning the two contig ends (see Figure 3.3 for genomic PCR results). Therefore, we concluded that this clone presents one allele with a clean c.1934T>G edit, whereas the other allele has a partial duplication of exon 6 and a ~ 5 kb plasmid insertion.

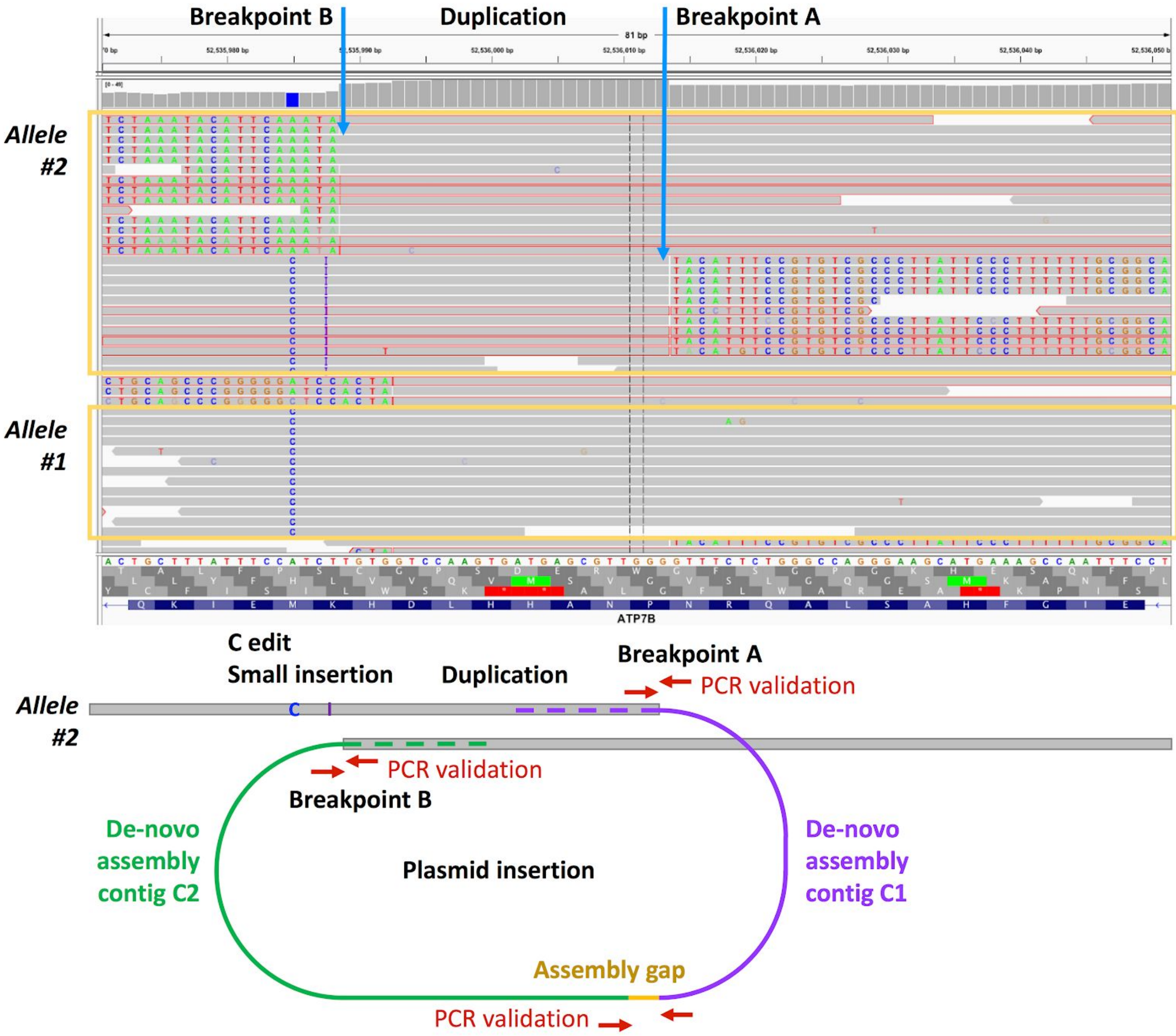

Figure 3.1 (same as supplementary figure 3): WGS results for the 2F3 HepG2 clone. Top: alignment to the human reference sequence, visualized using the Integrative Genomics Viewer (IGV), reveals three major read clusters, one corresponding to the edited c.1934T>G allele (allele 1) and the others suggesting a partial exon 6 duplication and plasmid insertion (allele 2). Bottom: the reconstructed sequence of allele 2, showing the human genome reference sequence as gray blocks, the contigs obtained by de-novo assembly as green and violet lines, the assembly gap as a gold line, the PCR primer sets used for validation as red arrow pairs; note that the plasmid insertion length is not proportional to the exon 6 length and that the de-novo assembly contigs span the exon 6 as well as nearby genomic reference sequence as suggested by the dashed lines.

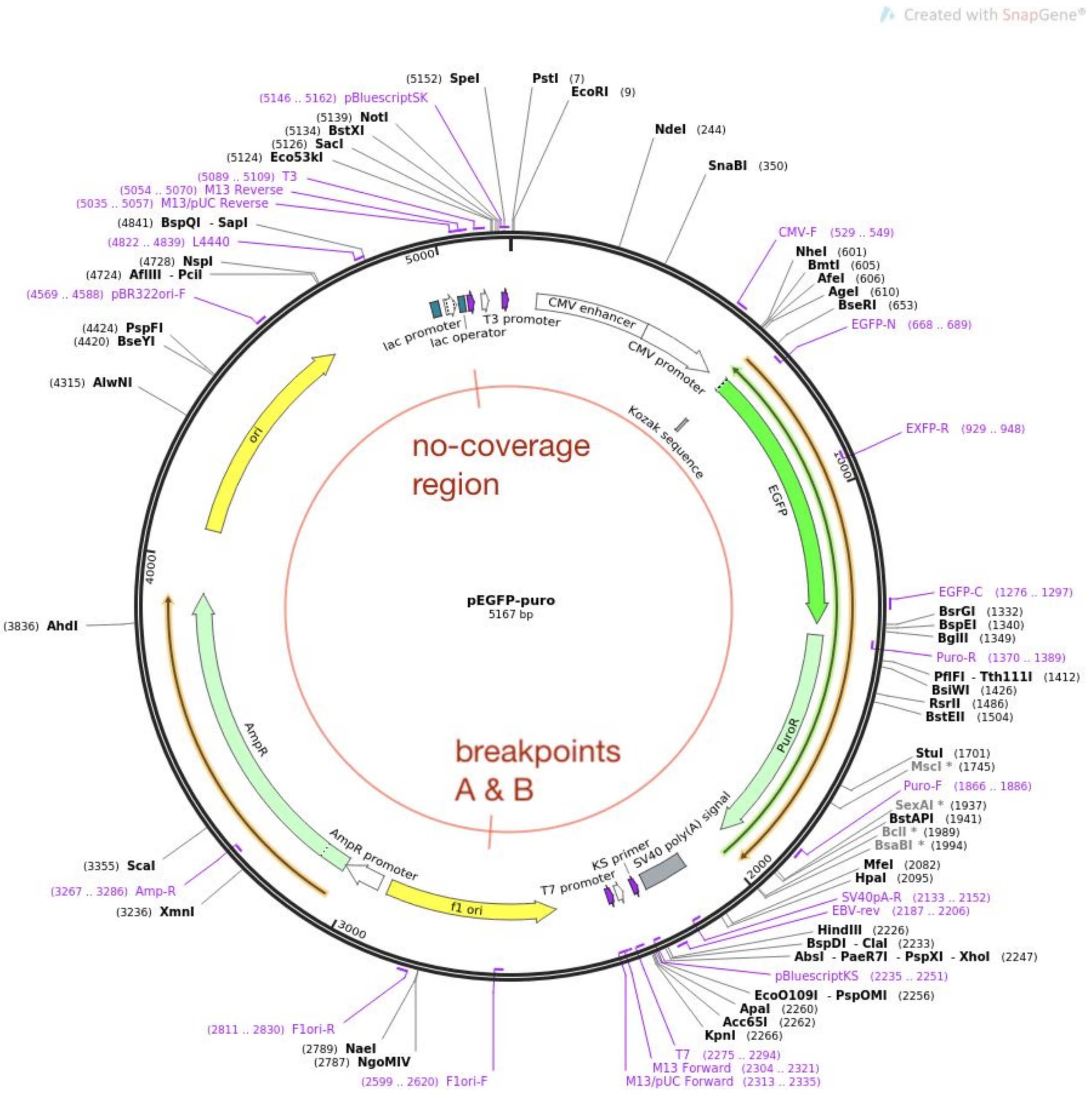

Figure 3.2: pEGFP-puro plasmid diagram, indicating the position of breakpoints A and B, and the “no coverage region” leading to the assembly gap after de-novo assembly.

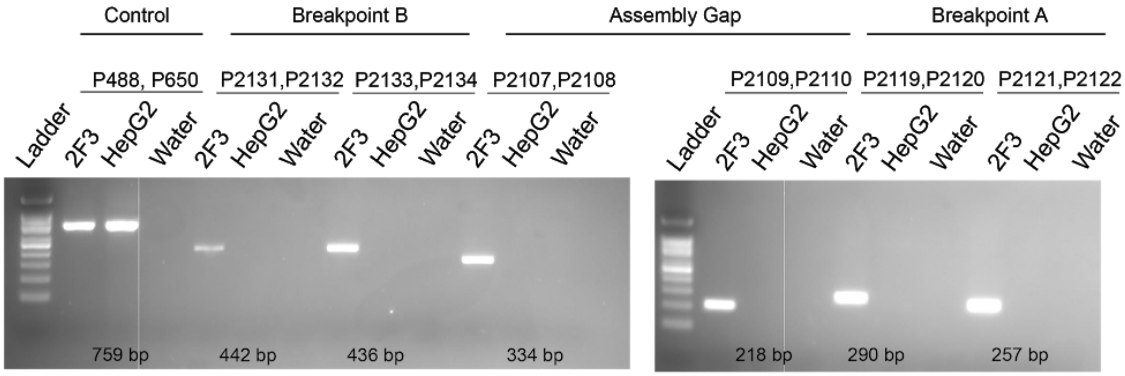

Figure 3.3: Genomic PCR covering Breakpoints A, B and assembly gap according to Figure 2 show amplification specifically in edited cells. Control PCR for ATP7B amplified in both edited and wild-type cells. Water serves as a negative template control.

(4) Frequency and Prevalence Calculations

Assuming that c.1934T>G is typically pathogenic only in compound heterozygosity with other pathogenic variants, and that Wilson Disease prevalence in Spain is the same as the consensus prevalence of 3.3 / 100,000, it is possible to estimate the c.1934T>G allele frequency in the general population of Spain, and verify if it is consistent with expectations, by solving the following equations.

Definitions:

- Frequency of c.1934T>G: Fv (to be estimated)
- Total frequency of ATP7B pathogenic variants: Ftot (to be estimated)
- Population of Spain: Spain_pop = 46.7 x 106
- Population of Spain with Wilson Disease: Spain_WD_pop = Spain_pop * WD_prevalence
- Prevalence of Wilson Disease: WD_prevalence = 3.3 x 10-5
- Fraction of Spanish WD patients with c.1934T>G: Spain_WD_var = 0.55

Equations:

- 2 * (Fv * (Ftot - Fv)) * Spain_pop = Spain_WD_pop * Spain_WD_var
- (Ftot ^ 2 - Fv ^ 2) * Spain_pop = Spain_WD_pop
- Spain_WD_pop = WD_prevalence * Spain_pop

Solving these equations results in the Spanish c.1934T>G frequency Fv = 0.002355 and total pathogenic variant frequency Ftot = 0.006209. Strikingly, this Spanish c.1934T>G frequency estimate is very similar to the c.1934T>G Latino frequency in gnomAD v2.1.1 (0.002233).
It is worth considering that the fraction of patients with c.1934T>G is subject to stochastic sampling error; we can repeat our calculation to estimate the frequency of c.1934T>G using instead the Clopper-Pearson 70% confidence interval (0.4559, 0.6412) of Spain_WD_var, which leads to Freq (c.1934T>G) = 0.001775-0.003075. Compared to the gnomAD v2.1.1 Latino frequency, these values appear to be reasonably in line with expectations.
In conclusion, c.1934T>G is present at a much higher frequency in Wilson Disease patients of Spanish descent and its allele frequency in the general population of Spanish descent is consistent with the consensus prevalence of Wilson Disease.

The depletion of homozygotes in the Margarit et al 2005 study can be assessed using the binomial test:

- Total patient count: snTot = 40
- Total allele count: anTot = 40 x 2 = 80
- c.1934T>G allele count: anVar = 22
- c.1934T>G allele frequency: afVar = anVar / anTot = 22 / 80
- Expected number of homozygous patients: snHom_e = afVar ^ 2 * snTot = 3.025
- Observed number of homozygous patients: snHom_o = 0

- Probability of encountering a homozygous patient: spHom = snHom_e / snTot = 0.075625
- One-sided binomial test p-value with alternative hypothesis that the observed number of homozygous patients is lower than expected (spHom): 0.04305[2]

Therefore we can conclude that there is a significant depletion of c.1934T>G homozygotes.

[2] binom.test (x = 0, n = 40, p = 0.075625, alternative = "less")

Supplementary Datasets

- Supplementary Dataset 1: primer sequences
- Supplementary Dataset 2: cRNA and ssODN sequences used for CRISPR/Cas9 HepG2 editing, CRISPR/Cas9 in-silico off-target analysis results

References

[Rodriguez 2013] Rodriguez JM, Maietta P, Ezkurdia I, Pietrelli A, Wesselink JJ, Lopez G, Valencia A, Tress ML. APPRIS: annotation of principal and alternative splice isoforms. Nucleic Acids Res. 2013 Jan;41(Database issue):D110-7. doi: 10.1093/nar/gks1058. PMID: 23161672

[GTEx 2013] GTEx Consortium. The Genotype-Tissue Expression (GTEx) project. Nat Genet. 2013 Jun;45(6):580-5. doi: 10.1038/ng.2653. PMID: 23715323

[Petrukhin 1994] Petrukhin K, Lutsenko S, Chernov I, Ross BM, Kaplan JH, Gilliam TC. Characterization of the Wilson disease gene encoding a P-type copper transporting ATPase: genomic organization, alternative splicing, and structure/function predictions. Hum Mol Genet 1994. PMID: 7833924

[Yang 1997] Yang XL, Miura N, Kawarada Y, Terada K, Petrukhin K, Gilliam T, Sugiyama T. Two forms of Wilson disease protein produced by alternative splicing are localized in distinct cellular compartments. Biochem J 1997. PMID: 9307043
